# Implementing advance care planning in routine nursing home care: The development of the theory-based ACP+ program

**DOI:** 10.1371/journal.pone.0223586

**Published:** 2019-10-17

**Authors:** Joni Gilissen, Lara Pivodic, Annelien Wendrich-van Dael, Chris Gastmans, Robert Vander Stichele, Liesbeth Van Humbeeck, Luc Deliens, Lieve Van den Block

**Affiliations:** 1 End-of-life Care Research Group, Vrije Universiteit Brussel (VUB) & Ghent University, Brussels, Belgium; 2 Centre for Biomedical Ethics and Law, Katholieke Universiteit Leuven (KUL), Brussels, Belgium; 3 Department of Pharmacology, Ghent University, Gent, Belgium; 4 Department of Geriatric Medicine, Ghent University Hospital, Gent, Belgium; 5 Department of Public Health and Primary Care, Ghent University, Gent, Belgium; 6 Department of Family Medicine and Chronic Care, and Department of Clinical Sciences, Vrije Universiteit Brussel (VUB), Brussels, Belgium; Beatrix Children's Hospital, University Medical Center Groningen, NETHERLANDS

## Abstract

**Background:**

While various initiatives have been taken to improve advance care planning in nursing homes, it is difficult to find enough details about interventions to allow comparison, replication and translation into practice.

**Objectives:**

We report on the development and description of the ACP+ program, a multi-component theory-based program that aims to implement advance care planning into routine nursing home care. We aimed to 1) specify how intervention components can be delivered; 2) evaluate the feasibility and acceptability of the program; 3) describe the final program in a standardized manner.

**Design:**

To develop and model the intervention, we applied multiple study methods including a literature review, expert discussions and individual and group interviews with nursing home staff and management. We recruited participants through convenience sampling.

**Setting and participants:**

Management and staff (n = 17) from five nursing homes in Flanders (Belgium), a multidisciplinary expert group and a palliative care nurse-trainer.

**Methods:**

The work was carried out by means of 1) operationalization of key intervention components–identified as part of a previously developed theory on how advance care planning is expected to lead to its desired outcomes in nursing homes–into specific activities and materials, through expert discussions and review of existing advance care planning programs; 2) evaluation of feasibility and acceptability of the program through interviews with nursing home management and staff and expert revisions; and 3) standardized description of the final program according to the TIDieR checklist. During step 2, we used thematic analysis.

**Results:**

The original program with nine key components was expanded to include ten intervention components, 22 activities and 17 materials to support delivery into routine nursing home care. The final ACP+ program includes ongoing training and coaching, management engagement, different roles and responsibilities in organizing advance care planning, conversations, documentation and information transfer, integration of advance care planning into multidisciplinary meetings, auditing, and tailoring to the specific setting. These components are to be implemented stepwise throughout an intervention period. The program involves the entire nursing home workforce. The support of an external trainer decreases as nursing home staff become more autonomous in organizing advance care planning.

**Conclusions:**

The multicomponent ACP+ program involves residents, family, and the different groups of people working in the nursing home. It is deemed feasible and acceptable by nursing home staff and management. The findings presented in this paper, alongside results of the subsequent randomized controlled cluster trial, can facilitate comparison, replicability and translation of the intervention into practice.

## Introduction

Advance care planning (ACP) is a process that supports adults at any age or stage of health in understanding and sharing their personal values, life goals, and preferences regarding future medical care [1,2]. If a person chooses, the contents of such conversations can be set down in writing [3].

Advance care planning is particularly relevant for frail older adults residing in nursing homes, due to the high probability that they will develop cognitive impairment and loss of decision-making capacity towards the end of life [4,5]. However, despite widespread recognition of its importance, still only a minority engaged in advance care planning [6,7]. Findings suggest this is the case for over a quarter of older US Medicare beneficiaries and the majority of long-term care residents [8,9]. In Europe, recent numbers show 32.5% of deceased residents had had a written directive, the most common type being a ‘do not resuscitate (DNR) order’. Extensive differences were found between countries [10]. A survey carried out in Flanders (Belgium) showed that a minority of deceased nursing home residents (11.8%) had expressed their wishes regarding end-of-life care, and that only 13.8% had a patient-reported advance directive at time of death [11]. For the purpose of documenting advance care planning, a number of possibilities are available in Belgium: an advance directive to refuse treatment (e.g. Do-Not-Resuscitate), nomination of a surrogate decision-maker and an advance statement which sets out general wishes or personal values. Only advance directives refusing treatment are legally binding for healthcare staff. Belgium also recognizes a type of positive advance directive for euthanasia [12–14]. To date, healthcare professionals in Belgium are not legally obliged to initiate advance care planning conversations with their patients but are encouraged to by local governments.

Recent reviews show advance care planning interventions, especially those in nursing homes, are increasingly multicomponent programs involving different types of staff training, education for patients and family, and elements such as flagging advance care planning outcomes in charts and feedback on a resident’s advance care planning status to physicians [15–17]. Researchers have stated with regard to this that nursing homes must change at every level, from management to frontline staff, if they are to achieve meaningful change in advance care planning uptake, and that such change should become and remain part of daily practice, not an on-off activity [18,19]. However, this is still what current advance care planning interventions often fail to do. They are mainly delivered by a ‘specialized group’ of expert facilitators [20], and training sessions are predominantly provided to nursing staff [21], social workers [22], and in rare cases, to healthcare professionals outside the facility (family physicians or emergency staff) [21]. Specific focus on engaging nursing home management and involving the entire nursing home workforce, including those that perform non-care tasks (e.g. cleaning staff or volunteers), has not been incorporated explicitly, although it is considered to be a crucial factor [23].

In previous work, we used a Theory of Change approach to develop a theoretical model of advance care planning for nursing homes [24]. This model is a ‘program theory’ rather than a ‘grand theory’ such as the Theory of Planned Behavior [25,26]. It shows how or under what circumstances advance care planning is hypothesized to work and can best be implemented in nursing homes in Flanders, Belgium. It outlines nine possible intervention components necessary to achieve change in the desired outcomes. However, these components need to be operationalized further into specific activities and intervention materials, tested for feasibility and acceptability, and described in such a way that they allow for comparison with other programs, replication, and translation into practice.

This paper reports on the development and description of the ACP+ program, a multi-component theory-based program that aims to implement advance care planning into routine nursing home care. The objectives of the study were threefold: 1) to specify how each intervention component can be delivered into routine nursing home care; 2) to evaluate feasibility and acceptability of the program; 3) to describe the final program in a standardized manner. The program is currently being evaluated in a cluster randomized controlled trial (ClinicalTrials.gov, no. NCT03521206, May 10, 2018).

## Methods and materials

The three objectives are achieved through three consecutive steps, outlined below. To develop and model our complex intervention according to the Medical Research Council (MRC) framework [27,28], we applied multiple study methods, including a literature review, discussions with a multidisciplinary expert group, semi-structured individual and group interviews with nursing home management and staff, and feedback from a palliative care nurse-trainer.

### Step 1. Translation of key intervention components into specific intervention activities and materials

The nine key intervention components, identified as part of a previously developed theoretical model on how advance care planning is expected to lead to its desired outcomes in nursing homes [24], are converted into specific activities with accompanying materials. To do so, we performed discussions within a multidisciplinary expert group and a review of existing advance care planning programs published in academic literature. The expert group consisted of an ethicist, three psychologists, a family physician, a sociologist, a social worker and a palliative care nurse who has a PhD in nursing and is specialized in providing training to healthcare professionals and implementing complex interventions in nursing homes. They convened once a month from April 2016 until March 2017. Available intervention materials from existing programs [21,22,29–39] were identified (e.g. training manuals, informational leaflets, conversation guide, documents), based on two existing systematic literature reviews and literature selection by the expert group [18,38,40]. The leading researchers in the two programs entailing a systematic, whole-setting approach and available in Dutch, were contacted to review the intervention materials they used for potential inclusion in our intervention [33,38]. For the intervention activities that we considered including in our intervention and for which no suitable materials could be identified in other existing programs, we used and adapted existing guidelines or informational materials, made available within the region (e.g. advance directives developed by the Belgian Federal Ministry of Health in 2017; www.leif.be) [29,41–43].

### Step 2. Evaluation of the feasibility and acceptability of the implementation of the program

We conducted an evaluation of the perceived feasibility (‘the extent to which the intervention can be delivered as intended’ [27]) and acceptability (‘the extent to which people delivering or receiving the intervention consider it to be appropriate’ [44]) of the intervention activities, the materials and the program’s implementation via interviews with nursing home management and staff, and revision of all intervention materials by the palliative care nurse-trainer.

1. Between April and November 2017, we carried out three semi-structured group interviews with 15 staff members and managers of three nursing homes, and two individual semi-structured interviews with healthcare professionals with extensive experience in advance care planning from two other nursing homes, because other team members in these nursing homes refused to participate due to busy work schedules. No additional interviews were carried out because we felt we had reached data saturation. The participants were paid nursing home employees and were recruited through convenience sampling via regional palliative care, dementia and nursing home networks and newsletters. Trainees and interns were excluded from participation. Each interview lasted on average 120 minutes (range: 90–190 min). All participants were asked to fill out a short survey of their individual characteristics (sex, age, job position, number of years active, training) and facility characteristics (type, number of beds, average number of deaths, guidelines available regarding palliative care, advance care planning documents, multidisciplinary meetings), and were asked to sign an informed consent form to audiotape the interview. All interviews were facilitated by JG and AWvD, according to a pre-specified topic list. Participants were asked to evaluate: (i) informational leaflets, guidance documents and manuals that we intend to use in the intervention, including those provided to participants or used in intervention delivery; (ii) enabling or supportive intervention activities; (iii) the modes of delivery of each intervention activity; (iv) any infrastructure and resources perceived necessary to deliver each intervention activity; (v) timing (including number of training sessions, advance care planning conversations, meetings), their schedule, and their duration; and (vi) which parts of the intervention should be adapted to better fit nursing home routine care. All audio records were transcribed.
2. All intervention materials were additionally reviewed and revised by and discussed with the nurse-trainer. She previously worked with the research team and was contacted directly by the researchers.

We applied thematic analysis to structure the comments of all participants, according to the Template for Intervention Description and Replication (TIDieR) checklist. Suggested adaptations were discussed within multiple meetings with the expert group and nurse-trainer. Decisions about changes to the initial intervention were consensus-based. Suggested changes that were not included in the renewed intervention, mainly due to time and resource constraints, are reported in S2 Table.

### Step 3. Standardized description of the final program according to TIDieR

To describe the final ACP+ program, we used the TIDieR checklist describing the why, who, how, where, when, how much and elements of tailoring of the intervention program [45].

## Results

### Translating ACP+ components into activities and materials (results of step 1)

Table 1 shows the original nine intervention components and the 16 intervention activities and materials underpinning them. The entire program and each of the activities should be implemented gradually, using a step-by-step approach. We distinguish a preparation phase and a follow-up phase. This phased implementation approach resulted from our previous work which built on theories highlighting that people and organizations progress through a series of stages or phases when modifying behavior or organizational structures with the help of interventions [24].

**Table 1:** ACP+ intervention components, intervention activities and materials (results of step 1, prior to evaluation of feasibility and acceptability).

Intervention component (n = 9)	Intervention activities (n = 16)	Intervention materials (n = 16)†
**1 |ACP (external) Trainer**	1. Selection and preparation of an (external) ACP Trainer, who provides adjusted support throughout stepwise implementation	1. Manual for ACP Trainer
**2 | Engagement/ Buy-in of management**	2. Meeting(s) between the ACP Trainer and the nursing home management, board of directors and coordinating advisory physician [46]*	2. ACP Information guide for nursing home management
**3 | ACP Reference Persons**	3. Selection of ACP Reference Persons 4. Two-day interactive training for the ACP Reference Persons, provided by the ACP Trainer	3. Training manual for two-day training
		4. ACP Manual for the ACP Reference Persons
**4 | Information about ACP**	5. Information (session(s)) for all care professionals, the coordinating advisory physician and the management 6. Information (session(s)) for all residents and their families about advance care planning and the policy/procedures in the nursing home	5. Invitation letter for staff, coordinating advisory physician and management for information sessions 6. Invitation letter for family physicians 7. ACP Information brochure for nursing home staff and family physicians 8. Invitation letter for residents and families 9. ACP information brochure for residents and family
	7. Information (session(s)) for all family physicians about advance care planning and the policy/procedures in the nursing home	
**5| Planned ACP conversations**	8. Exploration of previously recorded wishes and family physician involvement 9. First advance care planning conversation according to the ACP Conversation Guide, with resident and family or family alone, if resident is not able to participate 10. Follow-up advance care planning conversations (yearly or after trigger moments such as admission to hospital or death of a relative) 11. Documentation of wishes and preferences on a standardized form (of which a copy is saved in the resident’s file), a summary sheet and ADs (if perceived necessary by the resident, or family if resident is not able to participate)	10. ACP Conversation Guide 11. ACP Document 12. Standardized Advance Directive documents
**6 | In-house training**	12. In-house training sessions (session 1 and session 2) to train nurses (and others such as clerical workers, moral consultants, social workers, etc.) who are willing to conduct advance care planning conversations (called ACP Conversation Facilitators) 13. In-house training session to train other staff (care workers, hairdressers, cleaning staff, administrative staff etc.) and volunteers to train them to recognize triggers in residents and family (called ACP Antennas)	13. Training manual for training other staff
**7 | Multi-disciplinary meetings**	14. Multidisciplinary meetings are held and the advance care planning process for each resident is discussed (the resident’s most important decisions, possible triggers for initiating advance care planning with residents and/or family and discussions still planned)	14. Summary sheet
**8 | Reflection**	15. Reflective (debriefing) sessions among all care professionals at the nursing home in which they discuss the death and advance care planning process of every resident who died during that month	15. Reflection instrument
**9| Formal monitoring system**	16. A formal monitoring system is put in place in which the nursing home evaluates advance care planning organization and procedures	16. Audit instrument

ACP advance care planning.

*Nursing homes are legally obliged to have at least one coordinating and advisory physician (remunerated according to the number of beds), who coordinates medical care in the facility, as well as reference nurses for palliative care (0.10 FTE per 30 residents).

†The source of and adaptations made to every intervention material is reported in the Supporting Information Materials (S1 Table).

We also distinguish several roles. **ACP Trainers** will be available for nursing homes to support staff in implementing advance care planning. These trainers should be skilled and experienced in change management, have clinical practice experience in nursing homes and specifically in performing advance care planning conversations, and be able to train other professionals. The trainer’s support is intensive at the beginning but decreases throughout the process as the ACP Reference Persons become increasingly autonomous. The nomination of several **‘ACP Reference Persons’** is at the core of the program. These are professionals employed by the nursing home who have roles in daily resident care (e.g. head nurses, team coordinators, nurses, palliative care reference persons, reference persons for dementia, psychologists, members of the palliative care team). The ACP Reference Persons’ main responsibility is to implement and sustain advance care planning within the nursing home. They market the program, communicate that it has a high priority, provide training to other staff, conduct advance care planning conversations, and perform regular monitoring of advance care planning procedures and outcomes within the nursing home. **‘ACP Conversation Facilitators’** are healthcare staff, who are—along with ACP Reference Persons—responsible for planning and performing regular advance care planning conversations with residents and family. All other nursing home staff who do not *necessarily* provide resident care but do have daily contact with residents or family (e.g. care assistants, hairdressers, cleaning staff, administrative staff, volunteers), are called **‘ACP Antennas’**. They recognize and signal triggers that are indicative of a person being ready or willing to engage in advance care planning.

All intervention materials, prepared to deliver the ACP+ program, their original source and adaptations made to the materials by the researchers, before testing in step 2, are provided in in the Supplementary Information Materials (S1 Table).

### Revisions to enhance the feasibility and acceptability of the program (results of step 2)

The characteristics of the participants in step 2 can be found in S3 Table. The majority of participants were female, had more than 15 years’ work experience in their current position, and were trained in palliative care. Participants included nurses, care assistants, social workers, a coordinating advisory physician, a physical therapist, and management (i.e. quality coordinator and head of resident care). They were employed in public or private non-profit nursing homes, with numbers of beds ranging from 80 to 360.

Participants’ perceptions of the feasibility and acceptability of the program’s implementation did not vary extensively. All professional stakeholders and the nurse-trainer agreed with the suggested benefits of ACP+ for the nursing home and most thought the program was worthwhile. While maintaining the core principles of the program, their comments resulted in several adjustments to the components, activities and materials. Details of the identified issues and subsequent changes are provided in the Supporting Information Materials (S2 Table).

#### Important changes to intervention components and activities

Involving family physicians in an intensive information session was deemed not feasible. In addition, participants felt the provision of general information via leaflets and posters very helpful and that sessions should be adapted to fit the physician’s working schedules.

*“Family physicians will come to your information session if it is organized late*, *after 5 p*.*m*. *and if you arrange accreditation”* (quality coordinator)

*“Make sure staff are trained to contact the physician to make sure he/she knows an ACP conversation is about to be organized but make sure staff does not wait before the physician takes the first step”* (coordinating advisory physician)

In the final program, staff are asked to contact family physicians to inform them about the new advance care planning procedures and ask them how they would like to be involved in their patient’s advance care planning. Family physicians should be invited for an accredited information session, organized by a trainer and the nursing home’s coordinating advisory physician, after 5 p.m.

Staff felt the program would be too time-intensive if several intervention activities were not combined into one activity. It was also recommended always to take lack of time and low staffing levels into account while organizing intervention activities.

*“Make sure you combine the information session with the training of recognizing signals; and do this during lunch or at a time when it does not take up too much time*. *Split one session of 4 hours into 2 of 2 hours; otherwise care is interrupted*.*”* (nurse)

The activity aimed at informing staff, the nursing home’s coordinating and advisory physician, and management was removed and replaced by word of mouth, internal meetings, folders/posters and training sessions to communicate information about advance care planning to personnel who are additionally trained in recognizing triggers. Moreover, management and the coordinating physician should be informed earlier, at the newly added ‘management engagement meeting(s)’.

Participants voiced the need for activities that specifically encourage management engagement and support (called ‘buy-in’) and a clause in the written participation agreement stating that staff would be guaranteed enough time to carry out program-related tasks. For this reason, additional management meetings were added to the program. They will be specifically asked to give selected ACP Reference Persons the necessary time and mandate to carry out their tasks. Management was asked to select at least two reference persons in each ward who are guaranteed 0.10% FTE (full-time equivalent) to spend on activities of the ACP+ program. This excludes three full workdays of training (training and comeback seminar) and advance care planning conversations with residents and family.

All participants and the nurse-trainer felt the program could only be incorporated into usual care if it allowed for enough tailoring of details, in a way that is compatible with current practice. The same applies to multidisciplinary meetings which are ideally organized monthly, but there might be other forms and types of team meetings that may function as a platform to discuss advance care planning and changes in preferences of residents. In addition, it was recommended that nursing homes that are performing structural changes to their organization should not be included in the study. This was added to the exclusion criteria in the subsequent trial.

*“Every nursing home has its own structure and it is important we have some freedom to for example arrange the information sessions according to the ways we know (e*.*g*. *family meetings*, *coffee gatherings*, *resident board…)”* (nurse)

*“If there are structural changes (e*.*g*. *renovations to the building) the implementation of such a new program is not compatible*. *In such times organizing advance care planning fades and primary attention of staff goes to daily nursing care*.*”* (coordinating advisory physician)

We added ‘tailoring meetings’ as a separate intervention component. These meetings are carried out at the start of the implementation and are organized between facility manager, head nurses and staff responsible for implementing the program. The goal of these meetings is to determine which intervention aspects are to be tailored. As a result of this addition, the total number of intervention components changed from nine to ten.

Participants felt there was a lack of clear profile description of who these ACP Reference Persons ought to be. They were thought to be needing some *maturity* and experience to carry out the tasks related to the function, to have regular contact with residents and family and be able to handle any resistance from staff. They should have a particular interest in end-of-life care and/or advance care planning and be sufficiently trained. They should be willing to carry out this function and have the mandate from the management to do so. Some participants argued they additionally should have some medical knowledge. Others felt that others, such as social workers, could function as ACP Reference Persons too.

*“And even if you have had sufficient training*, *this is not something you can learn in one year with a short training*. *You need to practice and have experience*.*”* (physical therapist)

Within the multidisciplinary expert group, we agreed on selection criteria which can be used to select ACP Reference Persons within the first management meetings, always in dialogue with the person him/herself. ACP Reference Persons are professionals employed by the nursing homes, who have responsibilities in daily nursing home care. They are preferably a nurse or head nurse, a member of the palliative care support team within the nursing home or another healthcare professional who is experienced or has some interest in advance care planning and communication about end-of-life care, who is enthusiastic and motivated, has sufficient organizational skills and is good at stimulating colleagues. A list for selecting ACP Reference Persons was added in the ‘ACP Information guide for the nursing home management’.

All participants felt the trainer should be familiar with the specific context and working routines of the nursing home.

*“Availability of a specialized trainer will motivate nursing homes to enroll in the subsequent study …”* (head of resident’s care)

*“But he/she should know how we work*.” (nurse)

A site visit/rotation at the start of the intervention was deemed by the nurse expert to be an important addition to the training component in order for him/her to become familiar with the way of working in each nursing home. This was defined as a half-day site visit (called ‘shadowing’), preferably during a morning shift.

Ongoing support, especially a ‘comeback seminar’ halfway through the implementation period of the program, was perceived to be necessary for trained staff to reflect on and present successes, challenges and overall experiences of the program along with staff from the other nursing homes. Staff also stipulated they would need additional information regarding advance care planning with people living with dementia. Also ‘continuity’ was frequently called upon and not knowing how to communicate wishes of residents to others to make sure all involved professionals are informed. Participants said they were worried that reflection sessions would take up too much time, although they were perceived as useful by all. It was suggested such reflection could also be integrated into other types of team meetings that already exist.

*“I would like some more information regarding how to estimate cognitive capacity”* (reference person palliative care)

“*It is important that the staff know how to communicate with other professionals to make sure these wishes that we discussed are eventually followed*, *also in crisis situations”* (nurse)

As a result, reflection sessions were broadened to encompass one-to-one coaching, a specialized training session about dementia and a specialization session focused on communication with and information transfer to other professionals (such as emergency staff or family physicians). Reflection sessions were made optional and the trainer will be instructed to stimulate staff to integrate this in existing meetings.

#### Important changes to the intervention materials

Revisions to the intervention materials included: 1) simplified language and better explanations of unfamiliar words, activities and learning points; and 2) clear descriptions of the objectives of the ACP+ program and its specific activities within each manual, leaflet or guidance document. The font in the ACP leaflet for residents and family was deemed to be too small, and some text was removed to improve readability. A short 1-page version (‘The ACP Conversation Tool’), that can be used during advance care planning conversations (as communication guidance rather than a checklist), was added, as well as a list where names of residents can be noted who are eligible for advance care planning and with whom conversations have been planned. In addition, a checklist was developed to inform trainers and management/staff about which procedures and materials cannot be tailored and should be standardized. All new materials were developed and reviewed by the research team and the nurse-trainer. The summary sheet to be used in multidisciplinary meetings was found to be redundant and was excluded, and materials to support reflection sessions were changed to optional. The total number of intervention delivery materials changed from 16 to 17.

### Standardized description according to TIDieR (results of step 3)

Table 2 describes each intervention component, its timing, any supporting or enabling activities, the mode of delivery (whether it is provided in a group, duo or individually), intervention providers and participants involved during each activity, and materials to support the implementation or organization. Elements eligible for tailoring are highlighted.

**Table 2:** Description of final intervention according to TIDieR: The ACP+ program (results of step 3).

Timing	Intervention component (n = 10)	What (intervention activities, procedures and processes) (n = 22)	How (mode of delivery and whether it is provided individually or in a group)	Who (the intervention provider(s) and participants)	Materials (resources/tools that support the intervention activities) (n = 17)
**3 months prior to start of program**	**1 | ACP Trainer**	Activity 1A: **Selection and preparation of two ACP (external) Trainers†.** The research team provides the ACP Trainers with the necessary information and training regarding the ACP+ intervention.	NA	1) research team 2) ACP Trainer who is employed by the research team (50% FTE) and who is external to the nursing homes	**1. A list of necessary competencies** will be made by the research team to use during the selection procedure and to assign a professional to become ACP Trainer. **2. "Manual for ACP Trainer"** highlighting key issues of the ACP+ intervention program and guiding the ACP Trainer in performing their tasks (such as training the ACP Reference Persons and supporting them and the nursing home in implementing ACP).
**month 1**	**2 | Buy-in and engagement of management**	Activity 2A: **Meeting(s) between the ACP Trainer and the nursing home management, representatives of the board of directors, head nurses and the Coordinating Advisory Physician.** At this meeting or series of meetings, the ACP Trainer explains the project and asks management for their (active) participation. This participation will include integrating ACP into the general policy of the nursing home and ensuring the ACP Reference Persons are appointed and able to spend time on their tasks to implement and organize the ACP+ intervention program and ACP in general, within the routine care. At this meeting, they put forward care professionals from among the nursing home staff as ACP Reference Persons‡) (preferably in consultation with the staff themselves)	in a group*	1) research team 2) ACP Trainer 3) management, board of directors, head nurses and coordinating advisory physician*	**3. "ACP Information guide for the nursing home management".** This information guide highlights the key issues and challenges of ACP, how the ACP^+^ intervention works, how it should be implemented, what everyone's role is and how they should carry out all the steps within the ACP+ intervention program. It also includes what should be the profile, necessary competencies and selection criteria for selecting this ACP Reference Persons are described for the management §.
month 1 to 4*	**1 | ACP Trainer**	Activity 1B: **'Shadowing'.** During the first four months, the trainer follows the selected ACP reference persons in their daily job to become familiar with the aspects related to the nursing home, certain routines and ACP-related activities that are already in place.	duo or in a group	1) ACP Trainer 2) ACP Reference Person(s)	None
month 1 to 4*	**3 | Tailoring**	Activity 3: **Tailoring-meeting(s) between ACP Reference Persons, management and important decision-makers** about how to implement the ACP+ program in routine care (e.g. planning of training sessions, specialization sessions etc.). During these meetings, the Trainer specifically looks at intervention activities that should be 'tailored' to the specific setting and makes sure all intervention activities are planned per schedule within existing routine care. These meetings run parallel with the training session of the ACP Reference Persons (Activity 4A).	in a group	1) Reference Persons with support of ACP Trainer 2) Management 3) Decision-makers (e.g. head residents' care, head nurses, quality coordinator)*	**4. “Tailoring Checklist”**: A list which includes questions to guide the tailoring meetings (such as “Are their ACP documents already available at the nursing homes?” “Are there any ACP procedures already in place?” etc.). For each intervention component and activity, a list is provided with the minimum of elements that should be held constant over all nursing homes and which elements can be adapted to each nursing home routines (indicated in this table with an asterisk).
**month 1**	**4 | ACP Reference Persons**	Activity 4A: **Two-day interactive training (session 1) for the ACP Reference Persons**, provided by the ACP Trainer, to train ACP Reference Persons in performing their tasks and responsibilities. Session 1 includes: 1. Information about ACP, 2. How to conduct planned ACP conversations (according to the ACP guidance document), 3. How to document wishes and preferences, 4. How to inform residents and family about ACP, 5. The ACP+ program and the responsibilities and tasks of an ACP Reference Person and how to fit this into routine care (‘tailoring’).	in a group	1) ACP Trainer 2) Selected ACP Reference Persons from all participating nursing homes	**5. "Training manual for two-day training"**. Training manual with educational materials for the ACP Trainer to use in the training for ACP Reference Persons **6. "ACP Manual for ACP Reference Persons"**. This manual includes all the materials that are relevant for the ACP Reference Persons to use in the implementation and organization of all intervention activities in the program, and how each intervention component can be tailored to the nursing home’s routine care. **7. “Summary list**” (first version): List on which the ACP Reference Persons note all residents and their loved ones who are eligible for an ACP conversation. This list provides an overview of who scheduled a planned ACP conversation and when. It is also used to follow up who has a conversation planned, who should be involved in this conversation and when it is planned.
**month 2**	**4 | ACP Reference Persons**	Activity 4A: **Two-day interactive training (session 2) for the ACP Reference Persons**. Session 2 includes: 1. How to train other staff in performing planned ACP conversations (according to the guidance document), 2. How to educate other staff and volunteers in recognizing triggers for ACP, 3. How to integrate ACP in multidisciplinary meetings, 4. Problems and solutions with how to integrate ACP+ program in routine care.	in a group	1) ACP Trainer 2) Selected ACP Reference Persons from all nursing homes	as above
**month 3**	**5 | Information about ACP**	Activity 5A: **Information (session(s)) for all residents and their families** about ACP and the ACP policy/procedures in the nursing home in a format that is 'tailored' to routines in the specific nursing home setting (e.g. resident/family council, individually, exceptional information session)	individually or in a group (max 10 per group)*	1) ACP Reference Persons, supported by ACP Trainer 2) all eligible, consented residents/proxies and their family	8. "Invitation letter for residents and family"*, inviting them to participate in ACP information sessions. 9. "ACP information brochure for residents and family"*, including brief information about ACP and trigger questions for advance care planning
**month 3**	**5 | Information about ACP**	Activity 5B: **Information session(s) for all family physicians** about ACP and the ACP policy within the nursing home, including motivating them to consider the wishes and preferences of their patients in (end-of-life) decision-making and to engage in ACP with their patients. They also get information about the ACP process and structure of ACP conversations, the ACP standardized document and the advance directive. Format: physicians are invited to an information session after 5 p.m., accreditation can be arranged.	in a group*	1) ACP Reference Persons supported by ACP Trainer 2) Coordinating advisory physician 3) Family physicians who have one (or several) patient(s) in the nursing home 4) research team to provide organizational support	10. “Invitation letter for family physicians”*, inviting them to participate in these ACP information sessions. 11. "ACP Information brochure for professionals"*, including brief information about ACP and example questions to start and engage in a conversation, that all staff and physicians can keep in their pockets to remind them what the signals are for the initiation of ACP and how they can indicate these signals to the ACP Reference Persons.
**month 3**	**6 | In-house training**	Activity 6A: **In-house 2-hour training sessions (session 1) to train ‘ACP conversation facilitators’** to conduct ACP conversations. Session 1 is to train them in: general conversation skills to engage in conversations about end-of-life care.	in a group (max 10)*	1) ACP Reference Persons, supported by ACP Trainer 2) nurses in the nursing home that are willing (selected by important decision-makers)* 3) other healthcare staff (e.g. social worker, physiotherapist, psychologist, members of palliative support team) who are willing (selected by important decision-makers)*	**12. "Training manual for ACP Reference Persons to train other staff".**
**month 4**	**6 | In-house training**	Activity 6A: **In-house 2-hour training sessions (session 2) to train ‘ACP Conversation Facilitators’.** Session 2 is to train them in: how to conduct ACP conversations with residents and/or their family according to the guidance document, and how to document such conversations.	as above	same as above	as above
**month 4**	**6| In-house training**	Activity 6B: **In-house 1.5-hour training session to train ‘ACP Antennas’** to educate them in how to recognize triggers in residents and families, so they are more willing to have spontaneous ACP conversations according to their competencies and know how to pass on information to other staff.	as above	1) ACP Reference Persons supported by ACP Trainer 2) Staff and volunteers	as above
**month 5–8**	**7| Planned ACP conversations**	Activity 7A: **Exploration of earlier wishes and family physician involvement.** The person responsible for the conversation with the resident checks whether existing records of previous (documented) wishes are available and contacts the physician as to whether and how they want to be involved in the ACP process of the patient (e.g. do they want to receive a call each time something changes in the ACP, do they want to be involved in the conversations, etc.). The family physician is also asked about their knowledge of existing records of previous (documented) wishes or if they had ever had an ACP conversation, and whether there are any family dynamics that the nursing home staff should be attentive to. Activity 7B: **First planned advance care planning conversation** according to the ACP conversation guide, with resident and family or family alone if resident is not able to participate. Activity 7C: **Follow-up conversation(s)**. Activity 7D: **Documentation of wishes and preferences** on a standardized form (of which a copy is saved in the resident’s file), a summary sheet and advance directives (if perceived necessary by the resident).	duo (including the family physician*)	1) One of the ACP Reference Persons or an ACP Conversation Facilitator, supported by ACP Trainer 2) Eligible (consenting) residents and/or their family	13. **"ACP Conversation Guide"** which provides information about initiating and preparing for ACP conversations. This conversation guide also includes a flash card that professionals can use during the conversation itself. **14. “ACP Conversation Tool”:** short A4 document that ACP facilitators can use to guide the conversation. 15. "ACP Document"* 16. "Standardized advance directive documents"
**month 5–8** (every month 1 MDO)*	**8 | ACP information transfer**	Activity 8: (Monthly*) multidisciplinary meetings. The ACP process of each resident (the most important decisions of the residents, possible signals for initiating ACP with residents and/or family and discussions still planned), are discussed during regular multidisciplinary meetings so that information is shared among professionals in the nursing home. Ideally the family physician of each resident is involved in these meetings. If not, the ACP Reference Persons gives them a call and sends the ACP documents and advance directives, if used.	in a group*	1) ACP Reference Persons supported by ACP Trainer 2) Care professionals who are involved in the care of the resident (including volunteers and the family physician)*	None
month 5–8*	**9 | Coaching**	Activity 9A: **One-to-one coaching**. During months 5, 6, 7 and 8, the ACP Reference Persons are available for all ‘ACP conversation facilitators’ for one-to-one coaching, including questions, advice, discussing difficult ACP conversations, etc. Each ACP Reference Person makes sure their colleagues are aware they can ask for this one-to-one coaching or makes sure this is scheduled structurally*.	duo*	1) ACP Reference Persons supported by ACP Trainer 2) all ‘ACP Conversation Facilitators’ (or others*)	None
**month 6**	**4 | ACP Reference Persons**	Activity 4B: **Come-back seminar** for all ACP Reference Persons, organized by the ACP Trainer.	in a group	1) ACP Trainer (supported by the research team) 2) ACP Reference Persons	None
**month 6**	**9 | Coaching**	Activity 9B: **In-house specialization session 1: Dementia**. These sessions are organized by the ACP Trainer for all staff that perform ACP conversations (both ACP Conversation Facilitators and ACP Reference Persons).	in a group*	1) ACP Trainer 2) ACP Reference Persons 3) ACP Conversation Facilitators	Extra: **“Guideline for healthcare professionals working with people living with dementia”** [42]
**month 6**	**2 | Buy-in and engagement of management**	Activity 2B: **Follow-up meetings between management, other decision-makers, ACP Reference Persons and the ACP Trainer**.	In a group	1) Quality coordinator or person responsible for quality-assurance in the nursing home 2) ACP Reference Persons, with support of ACP Trainer 3) Important decision-makers (e.g. head of residents’ care, head nurses)	None
**month 7**	**9 | Coaching**	Activity 9C: **In-house specialization session 2: Communication with other healthcare professionals (e.g. hospital, family physician).**	In a group*	same as above	To be made by ACP Trainer
**month 8**	**10| Audit**	Activity 10A: **ACP audit meeting(s)**. To enhance **ongoing monitoring**, the nursing home manager responsible for the regionally regulated quality indicators for nursing homes in Flanders* makes sure the ACP procedures, policy and processes are discussed yearly with all involved healthcare professionals, the coordinating advisory physician and the management to identify problems and discuss action plans to improve current situations if necessary.	in a group*	1) Quality coordinator or person responsible for quality-assurance 2) ACP Reference Persons, with support of ACP trainer 3) important decision-makers (e.g. head of residents’ care, head nurses) 4) Coordinating advisory physician	**17. "ACP audit instrument"** which can be used to support yearly audit meetings

ACP advance care planning; TIDieR template for intervention description and replication; NA not applicable

*These activities can be tailored to the specific routine care at each nursing home (e.g. number of participants, number of sessions, who is involved, planning etc.).

†The ACP Trainer has the following necessary competencies: experience as a coach or trainer and preferably (work) experience in a nursing home or knowledge of the nursing home setting; knowledge of and/or experience in general principles of advance care planning and related conversations with patients/residents and/or family. Tasks: (1) To give explanations about the ACP+ program to management and staff members; (2) To facilitate the development of a an advance care planning policy and to enhance 'tailoring' of specific elements of ACP+; (3) To facilitate the division of roles and responsibilities of staff members and ACP Reference Persons involved in the process within the nursing home; (4) To train the ACP Reference Person in the nursing homes; (5) To support ACP Reference Persons in training other staff members; (6) To give adjusted support throughout all phases of the stepwise implementation of ACP+ (e.g. support, providing a role model, feedback, advice etc.).

‡The ACP Reference Persons are professionals employed by the nursing homes and have roles/responsibilities in daily nursing home care. They are preferably a (head) nurse, a member of the palliative care support team within the nursing home or another healthcare professional who is experienced and has interest in advance care planning and communication about end-of-life care, who is enthusiastic and motivated, has sufficient organizational skills and is good at stimulating colleagues. These ACP Reference Persons will become responsible for implementing and sustaining the advance care planning culture in the nursing home (after training and support from the trainer). They are able to: (1) conduct and follow-up planned conversations with residents and their families according to the ACP Conversation Guide; (2) adapt conversations to the residents’ cognitive capacity; (3) inform others about advance care planning; (4) (initially with the support of the ACP Trainer) a. train nursing colleagues (or other suitable clinical staff) to conduct planned conversations according to the ACP Conversation Guide, and b. educate other staff and volunteers to recognize triggers for advance care planning; (5) organize face-to-face reflection; (6) integrate advance care planning (outcomes) of residents/family during multidisciplinary meetings.

§The number of ACP Reference Persons per nursing home (at least two 0.10 FTE’s per 30 beds) depends on the number of beds in the nursing home. A minimum of two ACP Reference Persons will be assigned per 30 beds, which is the average number of beds in one ward.

**Fig 1:**
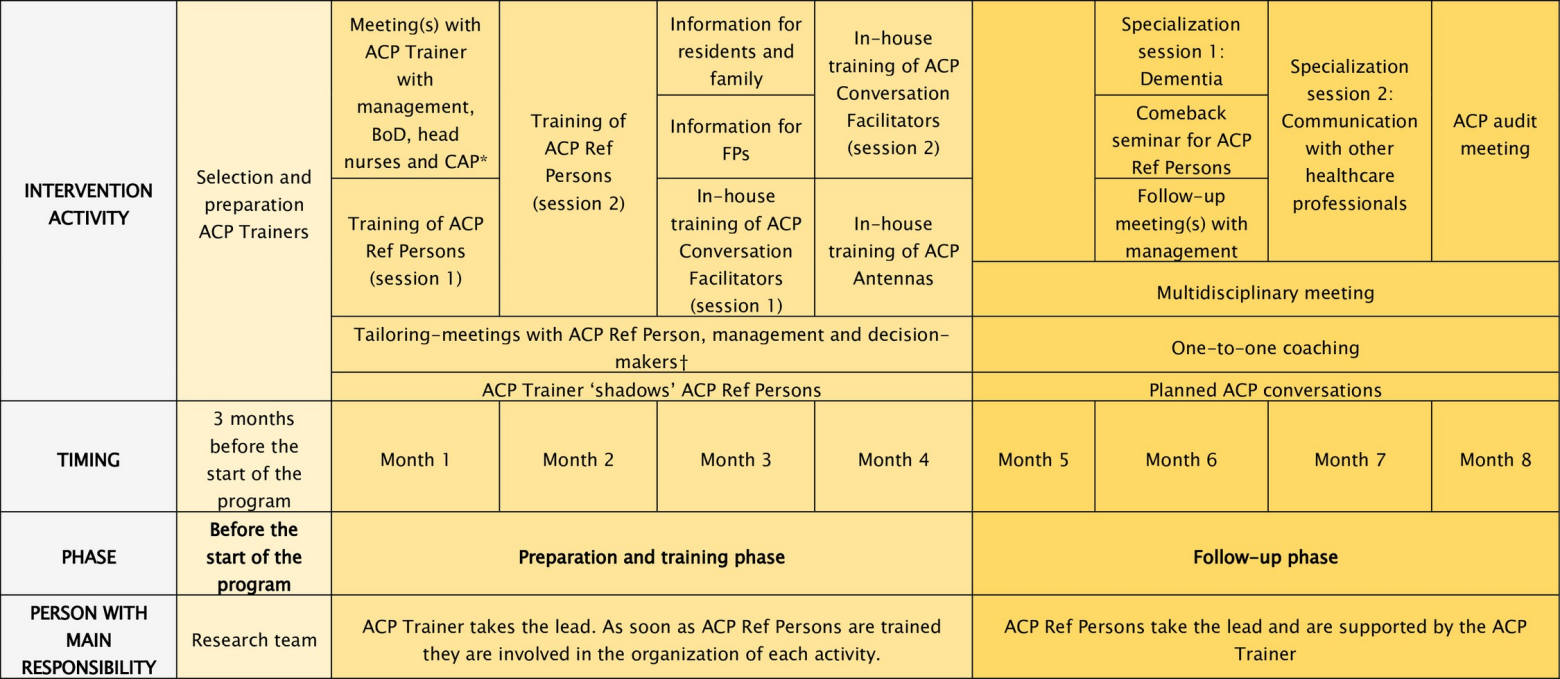
Timeline of the final ACP+ program. ACP advance care planning; BoD board of directors; CAP coordinating advisory physician; ACP Ref Person advance care planning reference person; FP family physician. The figure outlines the timeframe of the ACP+ program as how it will be evaluated in the subsequent trial. *Nursing homes are legally obliged to have at least one coordinating and advisory physician (CAP) (remunerated according to the number of beds), who coordinates medical care in the facility, as well as reference nurses for palliative care (0.10 FTE per 30 residents) [46]. †Important decision-makers include head of nursing staff, head of residents’ care, management; all those involved with decision-making tasks in the nursing home.

The entire program is carried out over eight months and consists of a preparatory training phase (months 1 to 4) and a follow-up phase (months 5 to 8). Fig 1 provides an overview of the timing of each activity and who is responsible. This timeline however is how we intend to implement the intervention in the subsequent trial and is therefore not strict and can be adapted in the future.

## Discussion

We present here the development and description of the ACP+ program, which is a comprehensive multicomponent and theory-based intervention that aims to implement advance care planning in nursing homes. The final program, which is described using the TIDieR checklist, consists of ten components ranging from training, coaching and management meetings, to planning advance care planning conversations, integration of advance care planning into multidisciplinary meetings and audit, all operationalized into 22 activities and 17 accompanying materials. These components are to be implemented stepwise over the course of at least eight months, with the help of an experienced trainer. Professional stakeholders perceived the ACP+ program to be feasible and acceptable for implementation in nursing homes in Flanders, if information sessions for family physicians were adapted, if enough tailoring was allowed, an experienced trainer who knows the nursing home context was available for coaching, comeback seminars and specialization sessions were organized (about dementia and communication with other healthcare professionals), and an additional specific focus on nursing home management’s buy-in was added to the program. In addition, simplified language in all intervention materials was advised. The final program focuses on creating both the necessary knowledge and attitudes and the underlying care ‘culture’ for successful advance care planning in nursing homes.

While there are some comparisons with other existing advance care planning programs (such as the educational train-the-trainer approach [21,33], the assignment of facilitators [21,30], the use of conversation guidance [30,47], informational materials and a standardized ACP document [33]) important differences remain. This intervention targets different levels in the facility, thus ensuring that implementation is not dependent on one individual but is embedded at organizational level [23]. The program also differs from others because it explicitly follows a stepwise approach (separating ‘preparation’ from ‘implementation’), in which the intensity of the trainer’s support decreases. Volunteers and cleaning or administrative staff in other programs had no explicit or specific role, despite research showing their importance in signaling care wishes of residents [48,49], but function as ACP Antennas in ours. Additionally, while there has been much emphasis on tailoring the initiation of advance care planning to patient readiness and willingness [50,51], and as both a process measure of implementation [28,45,52,53], there has been no explicit focus on the opportunity to tailor elements of advance care planning programs to suit local circumstances as part of the intervention itself. This is an important component of the ACP+ program.

## Strengths and limitations

The primary methodological strength of the reported research is the thorough process undergone to develop the intervention. Starting from a theoretical model [24,54], we operationalized and tested all components, activities and materials for their perceived feasibility and acceptability in the field. This work is in line with recent recommendations to start from theory and include testing feasibility and acceptability as part of the development phase of a complex intervention [27,55]. Step 2 (evaluating feasibility and acceptability) of our work provided the opportunity to identify implementation issues early on and to formulate strategies for these. This may minimize the need for modifications and the chance of implementation failure when testing the effectiveness of the intervention in a subsequent trial [56]. Second, by describing all details of this development work here, we comply with growing calls for more detailed and transparent reporting of complex healthcare interventions [45,55]. Our method has allowed us to provide a robust rationale for each foreseen intervention component, activity and material. As such, we believe this will enable researchers to compare our intervention with others more effectively, and practitioners to convert it more easily into clinical practice.

This study also has limitations. Firstly, we did not include the perspective of nursing home residents and their families when evaluating the feasibility and acceptability of the program. Hence, while the program is supported by a wide range of professional stakeholders, caution must be applied. Secondly, while we have put forward definitions of both feasibility and acceptability, it remains difficult to agree upon a cut-off point to decide when the intervention can be considered feasible or acceptable. Thirdly, because the intervention is adapted to the Flanders, some intervention components may not be directly transferable to other countries. Other countries may work with on-site physicians [57], or have better implemented electronic health records or different legal and financing systems [58,59]. Our advance care planning model involves intensive support of a specialized trainer at the start of the implementation; such resources might not be available everywhere. Finally, because project funding was time-limited, we did not carry out a pilot study e.g. a reduced version of the eight-month intervention program to determine whether the intervention components can all function well together [60]. However, we do aim to assess whether implementation of the program is worthwhile, whether it should be developed further or should be sent back to the drawing board [61], by using an in-depth process evaluation embedded in the subsequent trial.

## Conclusion and implications

ACP+ is a theory-based intervention program that aims to implement advance care planning in routine nursing home care. It consists of multiple components, activities and materials that need to be implemented together in a stepwise manner over the course of eight months with the help of an external trainer. Its thorough development process and the standardized description in this paper aim to prevent implementation failure in real practice and increase transparency, comparison with other interventions and replication in the future. The program is currently under evaluation as part of a cluster randomized controlled trial.

## Declarations

### Ethics approval and consent to participate

This study was approved by the Ethical Committee of University Hospital Brussels (2017/31 B.U.N. 143201732133). Anonymity was assured by removing participant information that could lead to identification. All participants were asked verbally for their consent to the publication of anonymized data.

## Supporting information

S1 Table ACP+ intervention materials per component (results of step 1, prior to evaluation of feasibility and acceptability). ACP advance care planning; GSF Gold Standards Framework (www.goldstandardsframework.org) *PACE is an EU-funded project (FP7) evaluating the PACE Steps to Success intervention to improve palliative care in nursing homes (www.eupace.eu) †*LEIF* “Belgisch LevensEinde InformatieForum” (Dutch) or “Belgian information forum for end-of-life care issues” (English) is an initiative by the Belgian federal government which is issued to provide information about end-of-life (care) issues to the public and professionals (www.leif.be). In 2017, they made several leaflets available to inform both the public and professionals about advance care planning. They have also developed and distribute advance directive forms, which are supported by the Belgian Federal Ministry of Health. ‡*Pallialine* is an initiative by the Flemish Federation for Palliative Care, assigned to develop evidence-based palliative care guidelines for practice. §*KBS King Baudouin Foundation Belgium* is a public benefit organization (www.kbs-frb.be/eng). In 2011 they organized a nationwide campaign to promote “thinking earlier…about later”, which resulted in several publications available in Dutch and French about advance care planning, including a guideline for professionals which was developed by a multidisciplinary team of experts.
(DOCX)

S2 Table Changes, additions and removals made to the original intervention components (n = 10), activities (n = 22) and materials (n = 17) of the ACP+ program (results of step 2). ACP advance care planning *Nursing homes are legally obliged to have at least one coordinating and advisory physician (CAP) (remunerated according to the number of beds), who coordinates medical care in the facility, as well as reference nurses for palliative care. †ACP codes are A, B, C [in Dutch language]: ‘A’ stands for ‘to do everything,’ ‘B’ stands for ‘preservation of functions’, ‘C’ stands for ‘comfort care’. Changes that were suggested by the participants but were not integrated in the renewed intervention because of resource and time restraints, were: 1) more training capacity (one trainer that is available to the nursing home full-time); 2) longer period of implementation time; 8 months is perceived not to be enough to implement ACP in a nursing home; 3) new electronic system (or adaptations to the existing one) to integrate advance care planning more easily into medical file of the patient; 4) extra financial resources to make sure nursing staff has enough time to train others and meanwhile conduct advance care planning with residents.
(DOCX)

S3 Table Characteristics of participants in interviews regarding feasibility and acceptability of the program (step 2). ACP advance care planning; NH nursing home; NA not available *Missing n = 4 †Nursing homes from which participants were recruited in individual semi-structured interviews ‡Organizing authority types: public, private commercial or private non-profit. §Number of beds in the nursing home as acknowledged by RIZIV (Belgian national health insurance administration), excluding beds at daycare centers and beds for short stays.
Information provided by one of the participants; residents who died between September 2016 and September 2017. ¶Response options: No or Yes; if yes, weekly, monthly or yearly.
(DOCX)

S1 File Interview guide (in Dutch). S1_File_ Supplementary material _Interview guide_Dutch.
(PDF)

S2 File Interview guide (in English). S2_File_Supplementary material_Interview guide_English.
(PDF)

## Abbreviations

ACP: advance care planning
TIDieR: Template for intervention description and replication
