# Supplementary material for: Implementing advance care planning in routine nursing home care: The development of the theory-based ACP+ program
Source: PLoS One. 2019 Oct 17;14(10):e0223586. doi: 10.1371/journal.pone.0223586 (PMC6797173; doi:10.1371/journal.pone.0223586)
Supplement: S1 Table — ACP advance care planning; GSF Gold Standards Framework (www.goldstandardsframework.org) *PACE is an EU-funded project (FP7) evaluating the PACE Steps to Success intervention to improve palliative care in nursing homes (www.eupace.eu) †LEIF “Belgisch LevensEinde InformatieForum” (Dutch) or “Belgian information forum for end-of-life care issues” (English) is an initiative by the Belgian federal government which is issued to provide information about end-of-life (care) issues to the public and professionals (www.leif.be). In 2017, they made several leaflets available to inform both the public and professionals about advance care planning. They have also developed and distribute advance directive forms, which are supported by the Belgian Federal Ministry of Health. ‡Pallialine is an initiative by the Flemish Federation for Palliative Care, assigned to develop evidence-based palliative care guidelines for practice. §KBS King Baudouin Foundation Belgium is a public benefit organization (www.kbs-frb.be/eng). In 2011 they organized a nationwide campaign to promote “thinking earlier…about later”, which resulted in several publications available in Dutch and French about advance care planning, including a guideline for professionals which was developed by a multidisciplinary team of experts. (DOCX) [file pone.0223586.s001.docx]

**S1 Table. ACP+ intervention materials, their original source and adaptations made**

| **Intervention materials** (n=16) | **Original source** | **Adaptations made compared to original source** |
| --- | --- | --- |
| 1. Manual for ACP Trainer | Manual for Country Trainers (PACE*; GSF) | Content adapted to purpose of ACP+, keeping original structure |
| 1. ACP Information guide for nursing home management | Information guide for nursing home management (PACE) | Adapted to purpose of ACP+ keeping original structure |
| 1. Training manual for two-day training | Outline 2-day workshop (PACE)  Content developed by research team | Adapted to purpose of ACP+, keeping original structure |
| 1. ACP Manual for the ACP Reference Persons | Manual for Coordinators (PACE; GSF) | Content adapted to purpose of ACP+, keeping original structure |
| 1. Invitation letter for staff, coordinating advisory physician and management for information sessions | Invitation letter (PACE) | Content adapted to purpose of ACP+, keeping original structure |
| 1. Invitation letter for family physicians | Invitation letter (PACE) | Content adapted to purpose of ACP+, keeping original structure |
| 1. Invitation letter for residents and families | Invitation letter (PACE) | Content adapted to purpose of ACP+, keeping original structure |
| 1. ACP Information brochure for nursing home staff and family physicians | ACP brochure for professionals made available by LEIF† | Shortened and adjusted layout |
| 1. ACP information brochure for residents and family | ACP brochure for population made available by LEIF† | Shortened and adjusted layout |
| 1. ACP Conversation Guide | Guideline for ACP conversations' and 'Guideline for ACP with people with dementia' (Pallialine‡); ACP guideline, no. 12 (Royal College of Physicians, 2009); ACP guideline (KBS§) | Inspired by various sources and adjusted to purpose of ACP+ |
| 1. ACP Document | Korfage et al., 2015; ACP document (University Hospital Leuven); 'Looking and Thinking Ahead' document (PACE EUFP7); Advance Care Plan (Respecting Patient Choices, Silvester et al., 2013) | Inspired by various sources and adjusted to purpose of ACP+ |
| 1. Standardized Advance Directive documents | Nationally accepted advance directives approved by federal government (developed and distributed by LEIF†) | Not adjusted |
| 1. Training manual for training other staff | Developed by research team | Not applicable |
| 1. Summary sheet | Summary Sheet (PACE) | Adapted to purpose of ACP+, keeping original structure |
| 1. Reflection instrument | Reflective Debriefing Instrument (PACE; Hockley et al., 2014) | Adapted to purpose of ACP+, keeping original structure |
| 1. Audit instrument | Regional quality indicators made available by Flemish government; ACP audit tool (Ampe et al., 2015); ACP quality indicators (Sinuff et al., 2015); Audit data tool (PACE) | Inspired by various sources and adjusted to purpose of ACP+; regional quality indicators are kept the same but are supported by an example of how to calculate each quality indicator |

ACP advance care planning; GSF Gold Standards Framework (www.goldstandardsframework.org)

**PACE* is an EU-funded project (FP7) evaluating the PACE Steps to Success intervention to improve palliative care in nursing homes [33] (www.eupace.eu)

†*LEIF* “Belgisch LevensEinde InformatieForum” (Dutch) or “Belgian information forum for end-of-life care issues” (English) is an initiative by the Belgian federal government which is issued to provide information about end-of-life (care) issues to the public and professionals (www.leif.be). In 2017, they made several leaflets available to inform both the public and professionals about advance care planning. They have also developed and distribute advance directive forms, which are supported by the Belgian Federal Ministry of Health.

‡*Pallialine* is an initiative by the Flemish Federation for Palliative Care, assigned to develop evidence-based palliative care guidelines for practice.

§*KBS King Baudouin Foundation* *Belgium* is a public benefit organization (www.kbs-frb.be/eng). In 2011 they organized a nationwide campaign to promote “thinking earlier…about later”, which resulted in several publications available in Dutch and French about advance care planning, including a guideline for professionals which was developed by a multidisciplinary team of experts [36].
