# Supplementary material for: Implementing advance care planning in routine nursing home care: The development of the theory-based ACP+ program
Source: PLoS One. 2019 Oct 17;14(10):e0223586. doi: 10.1371/journal.pone.0223586 (PMC6797173; doi:10.1371/journal.pone.0223586)
Supplement: S2 Table — ACP advance care planning *Nursing homes are legally obliged to have at least one coordinating and advisory physician (CAP) (remunerated according to the number of beds), who coordinates medical care in the facility, as well as reference nurses for palliative care. †ACP codes are A, B, C [in Dutch language]: ‘A’ stands for ‘to do everything,’ ‘B’ stands for ‘preservation of functions’, ‘C’ stands for ‘comfort care’. Changes that were suggested by the participants but were not integrated in the renewed intervention because of resource and time restraints, were: 1) more training capacity (one trainer that is available to the nursing home full-time); 2) longer period of implementation time; 8 months is perceived not to be enough to implement ACP in a nursing home; 3) new electronic system (or adaptations to the existing one) to integrate advance care planning more easily into medical file of the patient; 4) extra financial resources to make sure nursing staff has enough time to train others and meanwhile conduct advance care planning with residents. (DOCX) [file pone.0223586.s002.docx]

**S2 Table. Changes, additions and removals made to the original intervention components (n=10), activities (n=22) and materials (n=17) of the ACP+ program (results of step 2)**

| Intervention component and underlying activities | Changes/deletions made (reasons) | Newly added (reasons) |
| --- | --- | --- |
| **1 ACP TRAINER** |  |  |
| Selection and preparation | The external ACP Trainer is made available for only 8 months; hence no consolidation phase is included in the project (because of limited funding); A list with criteria to select an appropriate trainer was made available; Two trainers will be selected, each responsible for training a certain number of nursing homes |  |
| Shadowing |  | Shadowing for at least half a day was added for the trainers to get acquainted with the activities related to routines and ACP at each NH. No supporting materials deemed necessary. |
| **2 BUY-IN AND ENGAGEMENT OF MANAGEMENT** | | |
| Meetings with management, board of directors and coordinating advisory physician* | The meetings now Include management, all members of palliative care team/working group and head nurses; board of directors will not participate in these meetings |  |
| Follow-up meetings |  | Several meetings should be planned at the start and end of the study (Engagement of management is perceived as important step to guide implementation and sustainability) |
| **3 TAILORING** |  |  |
| Tailoring meetings |  | This was added as a new intervention component (because 95.1% of nursing homes in Flanders have ACP procedures in place [60]); A tailoring checklist is made available to the trainers and ACP Reference Persons (to know which elements should and cannot be standardized) |
| **4 TRAINING OF ACP REFERENCE PERSONS** | | |
| Training of ACP Reference Persons | Necessary number of ACP Reference Persons changed from 0.50 FTEs per 30 beds to two 0.10 FTEs per 30 beds (more feasible and sustainable in the long run); Content of training was adapted (to fit new ACP+ program) | Added as new training activity (Perceived important by all professional stakeholders to add this activity) |
| Come-back seminar |  |  |
| **5 INFORMATION** |  |  |
| Information about ACP for staff | Removed (because the suggestion was made to focus primarily on word of mouth/internal meetings, folders/posters and training sessions to communicate information about ACP to personnel; management and coordinating advisory physician should already have been informed at the management engagement meetings) |  |
| Information about ACP for family physicians | Timing changed from a session in the afternoon to a session in the evening (because patient visits are mainly before 5 p.m. which would guarantee more attendance); Accreditation must be guaranteed (to stimulate physicians to participate) |  |
| Information about ACP for residents and family | Both information sessions and additional information channels (e.g. in-house newspaper or letter, family gatherings, posters in elevator) are put to use, depending on availability in nursing home (To reach all residents and family members) |  |
| **6 IN-HOUSE TRAINING** |  |  |
| ‘In-house’ training to perform ACP conversations | Not limited to nursing staff, but also including others those who are members of the palliative care team, reference persons for dementia/palliative care, experienced care assistants (unsustainable due to staff turnover and limited nursing staff; on average 1 FTE nurse for 30 beds in Flanders); Changed to two sessions of 2 hours instead of one session of 4 hours (because this would take them away from their care duties for less time during the day); People who are trained to perform ACP conversations are called ‘ACP Conversation Facilitators’ |  |
| Info/training for other staff to signal ACP triggers | These training sessions Include all those who did not attend the previous training. Short sessions of 1.5 hours are enough. People trained to recognize signals are called ‘ACP Antennas’ |  |
| **7 ACP CONVERSATION PROCESS** |  |  |
| ACP conversations | An “ACP Conversation Tool” was added to the list of supporting materials. It is a short A4 document that ACP facilitators can use to guide the conversation (The “ACP Conversation Guide” was too elaborate; all stakeholders felt they needed a tool they could use during ACP conversations) |  |
| Follow-up of ACP conversations | The team should discuss every 6 months (or after trigger) if follow-up is necessary (challenging to revise all conversations yearly or after each trigger); A list should be made available to provide a summary of who has planned a conversation with which resident and who is eligible to participate |  |
| **8 INFORMATION TRANSFER** |  |  |
| Documentation | Tailoring is allowed (as it is bureaucratically and administratively challenging to implement new ACP documents; 4 out of 5 nursing homes have their own electronically available ACP documentation system); ABC codes are added to the ACP document (3 out of 5 nursing homes worked with this coding system†) |  |
| Multidisciplinary meetings | Perceived as underlying activity for information transfer |  |
| **9 COACHING** |  |  |
| Reflection sessions | We changed the name of this intervention component to ‘coaching’ and added ‘one-to-one coaching’ and two ‘specialization sessions’; The reflective debriefing instrument was kept as ‘optional’ |  |
| One-to-one coaching |  | Perceived by the professional stakeholders to be necessary after a difficult ACP conversation |
| Specialization session on dementia |  | This specialization session has been added (because nearly 2/3 of residents in participating nursing homes have mild to severe dementia) |
| Specialization session on communicating with others |  | This specialization session is added (because this was found difficult by nursing home staff) |
| **10 AUDIT** |  |  |
| Audit meetings | Changed from ‘formal monitoring system’ to the availability of an auditing tool (to guide a yearly meeting about ACP procedures); Because of the limited availability of quality coordinators in nursing homes, this intervention component was believed to be unsustainable after the study period. To develop a large monitoring system requires an in-depth literature review and development work, which was not possible due to the constraints of the research project) |  |

ACP advance care planning

*Nursing homes are legally obliged to have at least one coordinating and advisory physician (CAP) (remunerated according to the number of beds), who coordinates medical care in the facility, as well as reference nurses for palliative care [40].

†ACP codes are A, B, C [in Dutch language]: ‘A’ stands for ‘to do everything,’ ‘B’ stands for ‘preservation of functions’, ‘C’ stands for ‘comfort care’.

Changes that were suggested by the participants but were not integrated in the renewed intervention because of resource and time restraints, were: 1) more training capacity (one trainer that is available to the nursing home full-time); 2) longer period of implementation time; 8 months is perceived not to be enough to implement ACP in a nursing home; 3) new electronic system (or adaptations to the existing one) to integrate advance care planning more easily into medical file of the patient; 4) extra financial resources to make sure nursing staff has enough time to train others and meanwhile conduct advance care planning with residents.
