# Supplementary material for: Implementing advance care planning in routine nursing home care: The development of the theory-based ACP+ program
Source: PLoS One. 2019 Oct 17;14(10):e0223586. doi: 10.1371/journal.pone.0223586 (PMC6797173; doi:10.1371/journal.pone.0223586)
Supplement: S3 Table — ACP advance care planning; NH nursing home; NA not available *Missing n = 4 †Nursing homes from which participants were recruited in individual semi-structured interviews ‡Organizing authority types: public, private commercial or private non-profit. §Number of beds in the nursing home as acknowledged by RIZIV (Belgian national health insurance administration), excluding beds at daycare centers and beds for short stays. Information provided by one of the participants; residents who died between September 2016 and September 2017. ¶Response options: No or Yes; if yes, weekly, monthly or yearly. (DOCX) [file pone.0223586.s003.docx]

**S3 Table. Characteristics of participants in interviews regarding feasibility and acceptability of the program (step 2)**

| **Characteristics of participants (n=17)** |  |  |  |  |  |
| --- | --- | --- | --- | --- | --- |
| **Sex** | **Number** |  |  |  |  |
| Male | 3 |  |  |  |  |
| Female | 14 |  |  |  |  |
| **Age (years)*** |  |  |  |  |  |
| ≤ 29 | 1 |  |  |  |  |
| 30 – 39 | 2 |  |  |  |  |
| 40 – 49 | 4 |  |  |  |  |
| 50 – 59 | 6 |  |  |  |  |
| **Job position** |  |  |  |  |  |
| Social worker | 1 |  |  |  |  |
| Nurse | 3 |  |  |  |  |
| Head nurse/nursing unit manager | 3 |  |  |  |  |
| Head of resident care | 3 |  |  |  |  |
| Reference person for dementia | 1 |  |  |  |  |
| Care assistant | 2 |  |  |  |  |
| Reference person for palliative care | 1 |  |  |  |  |
| Physical therapist | 1 |  |  |  |  |
| Coordinating Advisory Physician | 1 |  |  |  |  |
| Quality coordinator | 1 |  |  |  |  |
| **Number of years active in current position** |  |  |  |  |  |
| < 7 years | 1 |  |  |  |  |
| 7 – 9 years | 2 |  |  |  |  |
| 10 – 15 years | 4 |  |  |  |  |
| ≥ 15 years | 10 |  |  |  |  |
| **Received training in advance care planning** |  |  |  |  |  |
| Yes | 15 |  |  |  |  |
| No | 2 |  |  |  |  |
| **Characteristics of participating nursing homes (n=5)** | **NH 1** | **NH 2**† | **NH 3** | **NH 4** | **NH 5**† |
| Organizing authority‡ | Private non-profit | Public | Public | Public | Private non-profit |
| Number of nursing care beds§ | 80 | 306 | 120 | 150 | 170 |
| Average number of residents who died in the past 12 months‖ | 33 | NA | 33 | 50 | 55 |
| Specific guidelines regarding palliative care practice available (yes/no) | yes | yes | yes | yes | yes |
| Patient-specific ACP documents available (yes/no) | yes | yes | yes | yes | yes |
| Multidisciplinary meetings regarding treatment and care plans of residents¶ | monthly | monthly | monthly | yearly | other  (every 5 weeks) |

ACP advance care planning; NH nursing home; NA not available

*Missing n=4

†Nursing homes from which participants were recruited in individual semi-structured interviews

‡Organizing authority types: public, private commercial or private non-profit.

§Number of beds in the nursing home as acknowledged by RIZIV (Belgian national health insurance administration), excluding beds at daycare centers and beds for short stays.

‖Information provided by one of the participants; residents who died between September 2016 and September 2017.

¶Response options: No or Yes; if yes, weekly, monthly or yearly.
