# Supplementary material for: Implementing advance care planning in routine nursing home care: The development of the theory-based ACP+ program
Source: PLoS One. 2019 Oct 17;14(10):e0223586. doi: 10.1371/journal.pone.0223586 (PMC6797173; doi:10.1371/journal.pone.0223586)
Supplement: S1 File — S1_File_ Supplementary material _Interview guide_Dutch. (PDF) [file pone.0223586.s004.pdf]

## TOPICLIJST (DUTCH)

**ALGEMENE VRAGEN OVER DE INHOUD VAN DE 'VZP INTERVENTIE (15 MINUTEN)**

1. Ten eerste zou ik willen dat je nadenkt over het werken met deze VZP interventie in zijn geheel. Straks zullen we meer gedetailleerd focussen op iedere fase en iedere stap. Wat is uw algemene impressie van de haalbaarheid van VZP+?
2. Is het haalbaar om dit binnen 8 maanden gedaan te krijgen? Wat is er nodig om het binnen 8 maanden gedaan te krijgen?
3. Wat vind je van de tijdspannes van de voorbereidingsfase, de implementatiefase en de consolidatiefase?
4. Is er voldoende tijd om de verschillende stappen uit te voeren?
5. Welke stappen hebben meer tijd nodig? Welke stappen kunnen met minder tijd toe?
6. Wat vindt u van de timing van de interventie (de start en einddatum)
7. Wat vindt u van de tijdsinvestering in de ACP-interventie?
8. Vind u dergelijke interventie zoals VZP+ gepast voor de omgeving van uw WZC?
9. Speciaal voor de studie werd er een VZP Trainer aangesteld. Wat vindt u hiervan? Welk profiel zou dergelijke trainer moeten hebben? Wat vindt u van zijn taakomschrijving?
10. Zien jullie de meerwaarde van dit programma voor jullie WZC?
11. Wat vindt u van de naam van dit VZP-interventie programma 'I PREFER', 'VZP+',…?

**VRAGEN OVER VZP INTERVENTIEMATERIAAL: "VZP HANDLEIDING VOOR VZP REFERENTIEPERSONEN IN WZC" (10 MINUTEN)** *- enkel met zorgpersoneel bespreken*

*Eerst hoor ik graag wat u vindt van de materialen dat wij u hebben doorgestuurd, zonder hier al op de inhoud van de 'i PREFER' interventie in te gaan.*

1. *Indien u geselecteerd werd om te fungeren als VZP Referentiepersoon, is het dan begrijpelijk wat hier staat? Snapt u direct wat er van u als VZP Referentiepersoon verwacht wordt?*
2. *Is het doel van dit VZP programma duidelijk in deze handleiding?*
3. *Is het gemakkelijk/duidelijk om te lezen?*
4. *Is het gemakkelijk om te zien wat er belangrijk is?*
5. *Is het gemakkelijk om te zien wat uw rol als VZP Referentiepersoon in elke stap en fase is?*
6. *Geeft dit document u voldoende handvatten om de taak uit te voeren? Wat kan er beter? Wat mist u nog?*

**VRAGEN OVER VZP INTERVENTIEMATERIAAL: "VZP INFORMATIEBROCHURE VOOR WZC PERSONEEL" (5 MINUTEN)** *- enkel met zorgpersoneel bespreken*

1. *Is het begrijpelijk wat hier staat? Snapt u direct wat er van u verwacht wordt?*
2. *Is het gemakkelijk/duidelijk te lezen?*
3. *Is het gemakkelijk om te zien wat er belangrijk is?*
4. *Is het doel van dit VZP programma duidelijk in deze informatiebrochure?*
5. *Geeft dit document u voldoende handvatten om de taak uit te voeren? Wat kan er beter? Wat mist u nog?*

**VRAGEN OVER VZP INTERVENTIEMATERIAAL: "VZP GESPREKSGIDS" (10 MINUTEN)** *- enkel met zorgpersoneel bespreken*

*1. Wat vindt u van het materiaal dat de gespreksvoerder krijgen ter ondersteuning van het voeren van een VZP gesprek ("VZP Gespreksgids")?*

*2. Wat vindt u van de documenten die gebruikt kunnen worden voor het documenteren van de*

*wensen en voorkeuren van de bewoner ("VZP Document 'Nadenken over Later'", "VZP Document: Samenvatting van wensen bewoner", "Wilsverklaringen LEIF")?*
*3. Is het begrijpelijk wat hier staat? Snapt u direct wat er van u verwacht wordt?*
*Is het gemakkelijk/duidelijk te lezen?*
*4. Is het gemakkelijk om te zien wat er belangrijk is?*
*5. Geeft dit document u voldoende handvatten om een VZP gesprek uit te voeren? Wat kan er beter? Wat mist u nog?*
*6. Geeft dit document u voldoende handvatten om wilsbekwaamheid in te schatten?*
*7. Geeft dit document u voldoende handvatten om wensen en voorkeuren betreffende zorg te documenteren?*

**VRAGEN OVER VZP INTERVENTIEMATERIAAL: "VZP AUDIT-INSTRUMENT" (10 MINUTEN)**
*1. Is het begrijpelijk wat hier staat? Snapt u direct wat er van u verwacht wordt?*
*2. Zijn de instructies duidelijk? Geeft dit document u voldoende handvatten om een eventuele audit uit te voeren?*
*3. Laten we elke indicator overlopen. Zijn ze allemaal duidelijk? Mist u nog iets? Is dit voldoende? Missen we hier nog iets?*
*2. Is elke indicator helder geformuleerd?*

**VRAGEN M.B.T. DE HAALBAARHEID EN GEPASTHEID I.F.V. DE UITVOERING VAN ELKE INTERVENTIE-ACTIVITEIT IN DE DAGELIJKSE PRAKTIJK (1u30min)**

Schrijf de inhoud van het VZP programma en de verschillende stappen op het bord (als er één beschikbaar is) of deel A4 bladen uit (zie bijlage “overzicht VZP interventie programma”). Deel hen ook een A4 blad met de TIDieR items uit (zie bijlage “overzicht te behandelen items per interventie-activiteit/stap”)**.** Voor elk van deze interventie-activiteiten (stappen) ga je na of er specifieke vragen, opmerkingen, hindernissen zijn betreffende de volgende TIDieR-items. Indien zij opmerkingen hebben, **vraag hen dan ook steeds hoe zij dit wel zien werken of wat dergelijke activiteit meer gepast zou maken.**

- ***Materialen***: De materialen die worden gebruikt/aangeboden per stap in het interventieproces.
- ***Procedures***: De activiteit en wat er per activiteit precies gaat gebeuren.
- ***Voor en door wie***: Door wie de interventie-activiteit worden georganiseerd/gedaan, met ondersteuning van wie en voor wie.
- ***Hoe georganiseerd (mode of delivery)***: Hoe de activiteit precies georganiseerd zal worden (face-to-face, in groep, individueel, elektronisch,...)
- ***Infrastructuur***: Welke infrastructuur er nodig is voor de activiteit (bijv. Een grote zaal, een kleine ruimte, een private gespreksruimte, een vergaderlokaal,...)
- ***Tijdstip/tijdsduur***: Wanneer de activiteit wordt georganiseerd, hoeveel en hoelang deze dan duurt.
- ***“Tailoring”:*** Wat er kan/moet aangepast worden door elk woonzorgcentrum. Waar zij ruimte zouden moeten krijgen om eigen aanpassingen te doen, naargelang hun eigen cultuur en werking binnen het woonzorgcentrum.

*We zullen nu verdergaan met de evaluatie van uw vragen, opinies en ervaringen met betrekking tot de VZP activiteiten en de verschillende stappen in het dagelijks gebruik.*

| **Deel 1** | |
|---|---|
| Interventie-activiteit | Vragen die de moderator kan stellen per TIDieR item (per interventie-activiteit) |
| **Meeting met management**<br>Geef kort uitleg | Materialen:<br>*1. Wat vindt u van het materiaal dat het management tijdens zal krijgen (“VZP Informatiefolder voor WZC Management”)?*<br>*2. Is het doel van dit VZP programma duidelijk in deze informatiebrochure?*<br><br>Procedures: |

| | |
|---|---|
| | *1. Wat vind u van deze activiteit?*<br>*2. Tijdens deze meeting zullen enkele personeelsleden geselecteerd worden om te fungeren als VZP Referentiepersoon. Is dit overleg het juiste format om dat te doen?*<br><br>Voor en door wie:<br>*1. Dit overleg wordt georganiseerd door een VZP Trainer (die werd aangesteld door het onderzoek), is dit de juiste persoon volgens u?*<br>*2. Tijdens dit overleg wordt het management, de hoofdverpleegkundigen en de CRA betrokken? Zijn dit de juiste mensen? Moeten er nog andere personeelsleden betrokken worden?*<br><br>Praktische organisatie:<br>*1. Wat vindt u van de praktische organisatie van deze meting? Wat werkt volgens u het beste?*<br><br>Infrastructuur:<br>*1. Is er een ruimte in uw WZC waarin dit overleg zou kunnen plaatsvinden?*<br><br>Tijdstip/tijdsduur:<br>1. *Dit overlegmoment wordt georganiseerd in de 1$^{e}$ maand. Wat vindt u hiervan?*<br>*2. Hoelang denkt u dat een dergelijk overleg duurt?*<br><br>"Tailoring":<br>*1. Moeten hier aanpassingen gebeuren, opdat dit beter haalbaar is? Opdat dit beter georganiseerd kan worden? Opdat dit beter aansluit bij het WZC?* |
| **Selectie en training van VZP Referentiepersonen**<br>Geef kort uitleg | Materialen:<br>*1. Voor de selectie van de VZP Referentiepersonen krijgen de deelnemers in de meeting met het management een criterialijst en lijst met competenties die hen kan helpen/ondersteunen bij de selectie van enkele personeelsleden (zie ("VZP Informatiefolder voor WZC Management"). Vindt u dit voldoende? Heeft u meer richtlijnen nodig? Wat mist u specifiek?*<br>*2. Deze VZP Referentiepersonen krijgen een tweedaagse training. Tijdens deze training krijgen zij een "VZP-handleiding voor VZP Referentiepersonen in WZC", inclusief alle documenten die er nodig zijn om de VZP-interventie zo goed mogelijk te implementeren. Wat vindt u hiervan?*<br><br>Procedures:<br>*1. Wat vindt u van de procedures van deze interventie-activiteit?*<br>*2. Wat vindt u van de procedures betreffende de selectie van de VZP Referentiepersonen?*<br>*3. Wat vindt u van de training van deze VZP Referentiepersonen?*<br><br>Voor en door wie: |

| | |
|---|---|
| | *1. Deze trainingen worden georganiseerd door de VZP Trainer. Is dit volgens u de aangewezen persoon?*<br>*2. Welk profiel/achtergrond moeten dergelijke VZP Referentiepersonen hebben?*<br>*3. Is 1 FTE per 30 bedden voldoende of te veel/te weinig?*<br><br>Praktische organisatie:<br>*1. Is dergelijke tweedaagse training haalbaar? Kunnen deze VZP Referentiepersonen hiervoor twee dagen vrijaf krijgen van hun dagelijks werk?*<br><br>Infrastructuur:<br>*1. Deze trainingen worden idealiter in het WZC zelf georganiseerd. Kan hiervoor een ruimte vrijgemaakt worden?*<br><br>Tijdstip/tijdsduur:<br>*1. Zijn de trainingen op het juiste moment (gedurende maand 2)?*<br>*2. Wat vindt u van twee dagen training? Te kort? Te lang?*<br><br>“Tailoring”:<br>*1. Zijn er zaken die, specifiek aan het WZC, aangepast zouden moeten worden?* |
| **Deel 2** | |
| Interventie-activiteit | Vragen die de moderator kan stellen per TIDieR item (per interventie-activiteit) |
| **Stap 1a: Informatiesessie voor personeel en huisartsen (30 min)**<br>Geef kort uitleg | Materialen:<br>*1. Wat vindt u van het materiaal dat het personeel en de huisartsen tijdens deze informatiesessies zullen krijgen (“VZP Informatiebrochure voor WZC personeel en huisartsen”, “Uitnodiging huisartsen”)?*<br><br>Procedures:<br>*1. Wat vindt u van de procedures van deze interventie-activiteit?*<br>*2. Is een informatiesessie volgens u de juiste manier om deze informatie over te brengen?*<br><br>Voor en door wie:<br>*1. Deze informatiesessie wordt georganiseerd door de VZP Trainer, samen met de VZP Referentiepersonen. Moeten hier nog iemand anders in betrokken worden?*<br>*2. Deze informatiesessie is voor <u>AL</u> het WZC personeel en alle huisartsen. Wat vindt u hiervan?*<br><br>Praktische organisatie:<br>*1. Zijn dergelijke informatiesessies haalbaar?*<br>*2. Kan het personeel hiervoor vrijaf krijgen van hun taken, gedurende een half uur?*<br>*4. Nadat u alles gehoord heeft, hoe zou u dit praktisch aanpakken?* |

| | |
|---|---|
| | *5. WZC personeel wordt op de hoogte gebracht door de VZP Referentiepersoon. Hoe hij/zij dat doet, kan hij/zij zelf beslissen. Wat lijkt u hiervoor de beste manier?*<br>*6. Huisartsen worden hiervoor via brief uitgenodigd. Is dit volgens u de beste manier?*<br><br>Infrastructuur:<br>*1. Deze informatiesessies worden idealiter in het WZC zelf georganiseerd. Kan hiervoor een ruimte vrijgemaakt worden?*<br><br>Tijdstip/tijdsduur:<br>*1. Gedurende de tweede maand worden deze informatiesessies georganiseerd. Idealiter zijn er dit twee. Eentje voor het personeel en huisartsen en eentje voor de bewoners/familie. Indien er weinig opkomst was, kan de VZP Referentiepersoon zelf beslissen om er meer te organiseren. Is dit haalbaar in een periode van 1 maand?*<br>*2. Deze informatiesessie duurt 30 minuten. Is dit voldoende? Te veel? Te weinig?*<br><br>“Tailoring”:<br>*1. Dergelijke informatiesessies kunnen misschien geïntegreerd worden in bestaande informatiesessies, familie- of bewonersraden of dergelijke,.. Wat denkt u hiervan? Welke zouden dit dan het beste zijn?*<br>*2. Hoe past dit in jullie dagelijkse zorgpraktijk?*<br>*3. Zijn er zaken die jullie zelf al doen en willen behouden? Zijn er zaken die aangepast moeten worden? Wat zijn jullie ideeën hierover?* |
| **Stap 1b: Informatiesessie voor bewoners en familie (30 min)**<br>Geef kort uitleg | Materialen:<br>*1. Wat vindt u van het materiaal dat de bewoners en hun naaste(n) tijdens deze informatiesessies zullen krijgen (“VZP Informatiebrochure voor WZC personeel en huisartsen”, “Uitnodiging familie/bewoners”)?*<br><br>Procedures:<br>*1. Wat vindt u van de procedures van deze interventie-activiteit?*<br>*2. Is een informatiesessie volgens jullie de juiste manier om deze informatie over te brengen?*<br><br>Voor en door wie:<br>*1. Deze informatiesessie wordt georganiseerd door de VZP Trainer, samen met de VZP Referentiepersonen. Moeten hier nog iemand anders in betrokken worden?*<br>*2. Deze informatiesessies worden georganiseerd voor alle bewoners en hun familie. Is dit haalbaar?*<br><br>Praktische organisatie:<br>*1. Zijn dergelijke informatiesessies haalbaar?*<br>*2. Is het haalbaar gedurende een half uur alle residenten en één van hun naaste(n) samen te krijgen?*<br>*3. Nadat jullie alles gehoord hebben, hoe zouden jullie dit praktisch* |

| | |
|---|---|
| | *aanpakken?*<br>*4. De bewoners werden via brief hiervan op de hoogte gebracht door de VZP Referentiepersoon. Wat vindt u hiervan? Hoe zou dit beter kunnen?*<br><br>Infrastructuur:<br>*1. Deze informatiesessies worden idealiter in het WZC zelf georganiseerd. Kan hiervoor een ruimte vrijgemaakt worden?*<br><br>Tijdstip/tijdsduur:<br>*1. Gedurende de tweede maand worden deze informatiesessies georganiseerd. Idealiter zijn er dit twee. Eentje voor het personeel en huisartsen en eentje voor de bewoners/familie. Indien er weinig opkomst was, kan de VZP Referentiepersoon zelf beslissen om er meer te organiseren. Is dit haalbaar in een periode van 1 maand?*<br>*2. Deze informatiesessie duurt 30 minuten. Is dit voldoende? Te veel? Te weinig?*<br><br>“Tailoring”:<br>*1. Dergelijke informatiesessies kunnen misschien ook geïntegreerd worden in bestaande informatiesessies, familie- of bewonersraden of dergelijke,.. Wat denkt u hiervan? Welke zijn dit?*<br>*2. Hoe past dit in jullie dagelijkse zorgpraktijk?*<br>*3. Zijn er zaken die jullie zelf al doen en willen behouden? Zijn er zaken die aangepast moeten worden? Wat zijn jullie ideeën hierover?* |
| **Stap 2: VZP gesprekken**<br>Geef kort uitleg | Materialen:<br>*Hier niet bespreken. Enkel in interview met zorgprofessionals.*<br><br>Procedures:<br>*Hier niet bespreken. Enkel in interview met zorgprofessionals.*<br><br>Voor en door wie:<br>*1. Deze VZP Gesprekken worden vanaf stap 2 reeds georganiseerd door de VZP Referentiepersonen. Na stap 3 zal ook het getrainde verplegend personeel VZP gesprekken doen. Wat vindt u hiervan?*<br>*2. Idealiter worden deze VZP Gesprekken gevoerd met iedereen die dat wenst. De inschatting van de wilsbekwaamheid is hierin natuurlijk een belangrijke stap. Toch zou elke bewoner betrokken moeten worden in dergelijk gesprek. Wat vindt u hiervan? Is dit haalbaar denkt u?*<br>*3. Idealiter wordt er ook steeds een familielid betrokken. De wettelijke vertegenwoordiger of, indien die er niet is, iemand waarmee de bewoner een vertrouwensrelatie heeft en wie in zijn plaats beslissingen mbt zijn zorg en behandeling kan nemen indien hij/zij dat zelf niet meer kan, zelf aangegeven door de resident. Indien de resident wilsonbekwaam is, is dit een familielid dat meest betrokken is bij de zorg van de bewoner. Wat vindt u hiervan? Is dit haalbaar denkt u?*<br><br>Praktische organisatie:<br>*1. De VZP Referentieperso(o)n(en) maken een lijst met bewoners en wie (bijv. welke getrainde verpleegkundige) met hen het gesprek moet* |

| | |
|---|---|
| | *aangaan. Zo is er een duidelijk overzicht wie welke gesprekken voert. Wat vindt u hiervan? Hoe kan dit beter?*<br>*2. Daarna nodigt de gespreksvoerder de bewoner en naaste(n) uit voor een VZP gesprek. Het is aan de gespreksvoerder zelf om te beslissen hoe hij/zij dit doet en om, in samenspraak met de resident en zijn/haar familie een follow-up gesprek in te plannen.*<br>*3. Telkens na een gesprek wordt idealiter het "VZP document: Nadenken over Later" ingevuld, en worden de belangrijkste beslissingen genoteerd op het "VZP document: Samenvatting", dat een gekleurd document is, en op een opvallende plek in het bewonersdossier wordt bewaard. Het is aan te raden hiervan nog eens een kopie op te sturen naar de huisarts en weerslag te nemen op de daarvoor bestemde plaats in het digitaal bewonersdossier. Is dit praktisch haalbaar? Hoe wordt dit praktisch het beste georganiseerd? Bijv. digitaal of op papier?*<br><br>Infrastructuur:<br>*1. Hebben jullie een stille ruimte/genoeg privacy om aan deze VZP gesprekken te doen?*<br><br>Tijdstip/tijdsduur:<br>*1. Vanaf stap 2 moeten deze gesprekken georganiseerd worden. Is dat haalbaar qua timing?*<br>*2. Indien de bewoner pas is opgenomen is dit binnen de 6 weken (max 12 weken) na opname. Is dit haalbaar qua timing?*<br>*3. Elk half jaar wordt er best eens opnieuw afgesproken met de bewoner en/of zijn familie. Is dit haalbaar?*<br><br>"Tailoring":<br>*1. Doen jullie nu ook al VZP in het WZC? Zo ja, hoe dan?*<br>*2. Zijn er zaken waaraan u denkt die best toch vrijgelaten worden aan de betrokken WZCs?* |
| **Stap 3a: Trainingssessie voor WZC personeel in het herkennen van signalen (1u30min)**<br>Geef kort uitleg | Materialen:<br>*1. Wat vindt u van het materiaal dat het WZC personeel tijdens deze informatiesessies zullen krijgen ("VZP Informatiebrochure voor WZC personeel en huisartsen")?*<br><br>Procedures:<br>*1. Wat vindt u van de procedures van deze interventie-activiteit?*<br>*2. Wat vindt u van dergelijke trainingssessie? Nuttig?*<br><br>Voor en door wie:<br>*1. Geschikt voor de zorgkundigen, schoonmaakpersoneel,…?*<br>*2. Alleen zorgkundigen die de training volgen?*<br>*3. Kunnen mensen hiervoor vrijgemaakt worden? Hoeveel tijd?*<br>*4. Zijn er andere zorgverleners waaraan u denkt die ook betrokken moeten worden in deze trainingen, opdat zij VZP gesprekken kunnen doen?*<br>*5. Deze training wordt georganiseerd en gegeven door de VZP Trainer, samen met de VZP Referentiepersonen. Wat vindt u hiervan?* |

| | |
|---|---|
| | *6. Idealiter in groepjes van 10 à 15 personen? Zou dit dan per functie opgedeeld moeten worden? Of net gemengd?*<br>*7. Gaat de VZP Referentiepersoon het personeel hiervoor kunnen motiveren? Wat met verplichten?*<br><br>Praktische organisatie:<br>*1. Het is aan de VZP Referentiepersonen om dit praktisch te organiseren. Zij krijgen hierin wel ondersteuning van de VZP Trainer. Hoe kan dit best georganiseerd worden volgens u?*<br>*2. De VZP Referentiepersoon zorgt dat al het personeel op de hoogte is van deze sessies, en zorgt ervoor dat iedereen de kans krijgt hieraan deel te nemen?*<br><br>Infrastructuur:<br>*1. Is hier een ruimte voor?*<br><br>Tijdstip/tijdsduur:<br>*1. Deze training duurt anderhalf uur. Te kort? Te lang?*<br>*2. Kunnen personeelsleden hiervoor vrijgemaakt worden?*<br><br>“Tailoring”:<br>*1. Hoe past scholing van personeel in jullie dagelijkse zorgpraktijk?*<br>*2. Doen jullie dit al? Zijn er zaken die aangepast moeten worden? Wat zijn jullie ideeën hierover?* |
| **Stap 3b: Training voor verpleging in het voeren van gesprekken (3 uur**)<br>Geef kort uitleg | Materialen:<br>*1. Wat vindt u van het materiaal dat de verpleegkundigen en vrijwilligers krijgen tijdens deze informatiesessies zullen krijgen (“VZP Informatiebrochure voor WZC personeel en huisartsen” en de “VZP Gespreksgids”, inclusief alle VZP documenten)?*<br><br>Procedures:<br>*1. Wat vindt u van de procedures van deze interventie-activiteit?*<br>*2. Wat vindt u van dergelijke trainingssessie? Nuttig?*<br><br>Voor en door wie:<br>*1. Geschikt voor verpleegkundigen? Allemaal? Hoeveel zijn dit er dan?*<br>*2. Geschikt voor vrijwilligers? Welke vrijwilligers? Hoeveel zijn dit er dan?*<br>*3. Kunnen verpleegkundigen hiervoor vrijgemaakt worden? Hoeveel tijd?*<br>*4. Zijn er andere zorgverleners waaraan u denkt die ook betrokken moeten worden in deze trainingen, opdat zij VZP gesprekken kunnen doen?*<br>*5. Deze training wordt georganiseerd en gegeven door de VZP Trainer, samen met de VZP Referentiepersonen. Wat vindt u hiervan?*<br>*6. Idealiter in groepjes van 10 à 15 personen? Zou dit dan per functie opgedeeld moeten worden: apart voor verpleegkundigen en vrijwilligers? Of net gemengd?*<br>*7. Gaat de VZP Referentiepersoon de verpleegkundigen en betrokken vrijwilligers hiervoor kunnen motiveren? Wat met verplichten?* |

| | |
|---|---|
| | Praktische organisatie:<br>*1. Het is aan de VZP Referentiepersonen om dit praktisch te organiseren. Zij krijgen hierin wel ondersteuning van de VZP Trainer. Hoe kan dit best georganiseerd worden volgens u?*<br>*2. De VZP Referentiepersoon zorgt dat al het verplegend personeel en de vrijwilligers op de hoogte is van deze sessies, en zorgt ervoor dat iedereen de kans krijgt hieraan deel te nemen. Hoe doet hij/zij dit best?*<br><br>Infrastructuur:<br>*1. Is hier een ruimte voor?*<br><br>Tijdstip/tijdsduur:<br>*1. Deze training duurt drie uur. Te kort? Te lang?*<br>*2. Kan verplegend personeel hiervoor vrijgemaakt worden?*<br><br>“Tailoring”:<br>*1. Hoe past scholing van zorgpersoneel in jullie dagelijkse zorgpraktijk?*<br>*2. Doen jullie dit al? Zijn er zaken die aangepast moeten worden? Wat zijn jullie ideeën hierover?* |
| **Stap 4: Reflectiesessies (1 keer om de 3 maanden)**<br>Geef kort uitleg | Materialen:<br>*1. Wat vindt u van het materiaal dat betrokkenen krijgen tijdens in de voorbereiding van deze reflectiesessies (“Reflectie-instrument”)?*<br><br>Procedures:<br>*1. Eén keer om de drie maanden wordt er een reflectiesessie georganiseerd over alle overleden bewoners van die drie afgelopen maanden. De VZP Referentiepersoon nodigt alle betrokken zorgverleners (die betrokken waren bij de zorg van deze overleden bewoners) uit. Wat vindt u van de procedures van deze interventie-activiteit?*<br>*2. Wat vindt u van dergelijke reflectiesessie? Nuttig?*<br><br>Voor en door wie:<br>*1. Deze reflectiesessies gaan over alle overleden bewoners van de afgelopen drie maanden. Hoeveel zijn dit er gemiddeld denkt u?*<br>*2. Deze sessies zijn voor alle betrokken zorgverleners (die betrokken waren bij de zorg van deze overleden bewoners). Wij denken hierbij vooral aan zorgverlenend personeel, maar ook vrijwilligers. Kunnen vrijwilligers hierin betrokken zijn? Ook als het gaat over bewoners die zij niet kenden?*<br>*3. Deze sessies worden georganiseerd door de VZP Referentiepersoon (in het begin, samen met de VZP Trainer). Wat vindt u hiervan?*<br><br>Praktische organisatie:<br>*1. De VZP Referentiepersoon staat vrij om te beslissen hoe deze reflectiesessies precies worden georganiseerd. Wat vindt u hiervan?*<br><br>Infrastructuur: |

| | |
|---|---|
| | *1. Dergelijke reflectiesessie gebeurt idealiter in een afgesloten ruimte waar plaats is om een overleg te plegen. Is die ruimte er?*<br><br>Tijdstip/tijdsduur:<br>*1. De reflectiesessies vinden plaats 1 keer om de drie maanden? Te veel? Te weinig?*<br>*2. Deze reflectiesessies duren +/- 1 uur. Deze tijdsduur kan variëren afhankelijk van het aantal overlijdens.*<br><br>“Tailoring”:<br>*1. Hoe past dit in jullie dagelijkse zorgpraktijk?*<br>*2. Doen jullie dit al? Zijn er zaken die aangepast moeten worden? Wat zijn jullie ideeën hierover? Zijn er overlegmomenten waarin dit geïntegreerd zou kunnen worden?* |
| **Stap 5: VZP integreren in maandelijkse multidisciplinaire overlegmomenten (MDO’s)**<br>Geef kort uitleg | Materialen:<br>*1. Wat vindt u van het materiaal dat VZP Referentiepersonen kunnen gebruiken om de andere zorgverleners op de hoogte te brengen van de belangrijkste (wijzigingen van) wensen en voorkeuren van de bewoner, wilsverklaringen, medische levenseindebeslissingen? (“VZP Document: samenvatting van wensen bewoner”)?*<br><br>Procedures:<br>*1. Wordt er maandelijks een MDO georganiseerd? Indien niet, zouden jullie dit dan organiseren?*<br>*2. Wat vindt u van dergelijke integratie van VZP in de MDO’s? Nuttig? Is dit mogelijk?*<br><br>Voor en door wie:<br>*1. Wie neemt in deze maandelijkse MDO’s deel?*<br>*2. Idealiter neemt de huisarts ook deel aan deze MDO’s?*<br>*3. Hoe brengt men de huisarts op de hoogte van de wensen en voorkeuren van de bewoner?*<br><br>Praktische organisatie:<br>*1. De VZP Referentiepersoon staat vrij om te beslissen hoe hij of zij deze wensen en voorkeuren in de MDO’s precies integreert. Wat vindt u hiervan? Hoe moet hij/zij dit aanpakken?*<br><br>Infrastructuur:<br>*1. Dergelijke MDO’s gebeuren idealiter in een afgesloten ruimte waar plaats is om een overleg te plegen. Is die ruimte er?*<br><br>Tijdstip/tijdsduur:<br>*1. Deze MDO’s worden idealiter elke maand georganiseerd. Is dit haalbaar?*<br>*2. Doet u MDO’s. Hoelang duren deze?*<br><br>“Tailoring”:<br>*1. Hoe past dit in jullie dagelijkse zorgpraktijk?* |

| | |
|---|---|
| | *2. Doen jullie al MDO's? Zijn er zaken die aangepast moeten worden? Wat zijn jullie ideeën hierover?* |
| **Deel 3** | |
| Interventie-activiteit | Specifieke hindernissen per TIDieR item |
| | Algemene vragen:<br>*1. Contactopname van VZP trainer (via telefoon, e-mail, langskomen,...)? Met wie?*<br>*2. Wanneer komt hij/zij dan best langs?* |
| **Stap 6: Audit (1 keer om het jaar)**<br>Geef kort uitleg | Materialen:<br>*1. Wat vind u van het materiaal dat VZP Referentiepersonen kunnen gebruiken om deze audit uit te voeren ("Audit-instrument")?*<br><br>Procedures:<br>*1. Wat vindt u van de procedures van deze interventie-activiteit?*<br>*2. Wat vindt u van deze audit? Nuttig?*<br><br>Voor en door wie:<br>*1. Deze audit wordt georganiseerd door de VZP Referentieperso(o)n(en), samen met de hoofdverpleging en de CRA. Moet hier nog iemand anders bij betrokken worden?*<br>*2. De resultaten van deze audit worden elk jaar teruggekoppeld naar het management en naar het betrokken personeel. Welk personeel is dit dan?*<br><br>Hoe georganiseerd:<br>*1. Deze audit wordt jaarlijks georganiseerd door de VZP Referentierpersoon. Het is aan hem/haar om te bepalen hoe hij/zij dit organiseert. Hoe vindt u dat dit georganiseerd moet worden?*<br><br>Infrastructuur:<br>*Geen*<br><br>Tijdstip/tijdsduur:<br>*1. Neemt dit veel tijd in beslag denkt u? Zo ja, hoeveel?*<br>*2. Kan de VZP Referentiepersoon hiervoor tijd vrijmaken?*<br>*3. Deze audit wordt jaarlijks herhaald. Is dit te vaak? Te weinig frequent?*<br><br>"Tailoring":<br>*1. Hoe past dit in jullie dagelijkse zorgpraktijk?*<br>*2. Doen jullie dit al? Zijn er zaken die aangepast moeten worden? Wat zijn jullie ideeën hierover?* |
