# Supplementary material for: Implementing advance care planning in routine nursing home care: The development of the theory-based ACP+ program
Source: PLoS One. 2019 Oct 17;14(10):e0223586. doi: 10.1371/journal.pone.0223586 (PMC6797173; doi:10.1371/journal.pone.0223586)
Supplement: S2 File — S2_File_Supplementary material_Interview guide_English. (PDF) [file pone.0223586.s005.pdf]

TOPICLIJST (ENGLISH)

Translated by first author (J. Gilissen)

GENERAL QUESTIONS REGARDING THE CONTENT OF THE ACP INTERVENTION (15 MINUTES)

We sent you a general outline of the envisaged intervention programme.

1. First of all, we would like you to think about working with this ACP intervention in general. In a next phase of the interview we will focus more in detail on every phase and step. What is your general appraisal of the feasibility of ACP+?
2. Is it feasible to go through the intervention within the time scope of 8 months? Wat is necessary to make sure it can be completed in 8 months.
3. What are your feelings regarding the timeframe of the preparation, implementation and consolidation phase?
4. Is there enough time to complete all steps of the intervention?
5. Which steps need more time? Which ones take less time?
6. What do you think about the timing of the intervention? Start and end date?
7. What do you think about the envisaged time-investment of staff in the ACP intervention?
8. Is this ACP+ intervention in its current form acceptable to implement in the environment of your nursing home?
9. Especially for the study we contracted a specialized trainer. What are your thoughts? Which profile should he/she have? What should be his/her tasks?
10. Do you feel this programme has an added value?
11. What are your thoughts regarding the name of this programme? ‘I Prefer’, ‘VZP+’…?

QUESTIONS RELATED TO ACP INTERVENTION MATERIAL: GUIDANCE DOCUMENT FOR ACP REFERENCE PERSONS 10 MINUTES _ *only discuss with care professionals*

We sent you the intervention materials.

1. If you were to be selected to function as an ACP reference person, would this guidance document be enough to function as a reference person? Is everything understandable? Is it clear what is expected of you?
2. What is the goal of the ACP+ program? Is it clear from the guidance document?
3. Is it easy/clear to read?
4. Is it easy to see what is found important?
5. Is it easy to see what your role is exactly? In every step and phase of the implementation?
6. Does this document provide you with enough guidance to carry out your task? What could be better? Which information you perceive necessary is missing?

QUESTIONS ABOUT INTERVENTION MATERIALS: “ACP INFORMATION LEAFLET FOR NURSING HOME STAFF” (5 MINUTES) *- only discuss this with care professionals*

We sent you the intervention materials.

1. Is it understandable?
2. Easy to read? Clear?
3. Easy to see what is important?
4. Clearly highlighted what is the goal of ACP? Why it is important?
5. Does this document give you enough guidance to carry out ACP? What can be better? What is missing?

QUESTIONS ABOUT INTERVENTION MATERIALS: "ACP CONVERSATION GUIDANCE DOCUMENTS" (10 MINUTES) – *-only discuss this with care professionals*

We sent you the intervention materials.

1. What are your thoughts about this document that should provide support to those that are performing ACP conversations?
2. What do you feel about the documents that can be used to document the wishes and preferences of the resident?
3. IS it understandable what says here? Do you understand what is expected from you?
4. Is it clear/easy to read?
5. Is it clear to see what is important?
6. Does this document give you enough support to carry out an ACP conversation? What can be better? What is it that you miss?
7. Does this document give you enough support to make an estimation of someone's cognitive capacity?
8. Gives this document enough support to document and how to document wishes and preferences regarding care and treatment?

QUESTIONS ABOUT INTERVENTION MATERIALS: "ACP AUDIT-INSTRUMENT" (10 MINUTEN)

We sent you the intervention materials.

1. Is it understandable what says here? Do you understand what is expected from you?
2. Is it clear/easy to read?
3. Is clear to see what is important?
4. Are the instructions clear? Does this document give you enough support to fulfil the audit?
5. Let's go through every indicator. Are they all clear? Do you miss something? Is this enough? What do we miss?
6. Is every indicator clearly formulated?

QUESTIONS RELATED TO THE FEASIBILITY AND ACCEPTABILITY WITH REGARDS TO THE IMPLEMENTATION AND ORGANISATION OF EVERY INTERVETION ACTIVITY IN DAILY PRACTICE (1 HOUR AND 30 MINUTES)

Instructions for researcher: Write down the content and each of the various steps of the programme on a board or share A4 papers to the participants. Also share with them each of the TIDieR items. For each of the intervention-activities consider specific questions, remarks and challenges for each of the TIDieR items. If they have suggestions, always ask them how they feel it will work and what would make the activity more acceptable in their setting.

Topic list structured according to TIDieR items

- Materials
- Procedures
- For and by whom
- How organised (mode of delivery)
- Infrastructure
- Timing/duration
- Tailoring (To what extent should this activity be adapted to their nursing home)

We will now carry on by your evaluation of each of the ACP intervention activities per step/phase.

**Part 1**	
Intervention-activity	Questions for moderator to ask per TIDieR item (per intervention-activity)
**Meeting with management**<br>Explain (in short)	Materials<br>*1. What are your thoughts regarding the materials for the management (information folder for nursing home management)?*<br>*2. Is the goal of the ACP programme clearly outlined in this material?*<br><br>Procedures<br>*1. What are your thoughts regarding this activity?*<br>*2. During this meeting some of the staff will be selected to function as ACP Reference person. Is such a meeting the right format to do so?*<br><br>For and by whom<br>*1. This meeting is organized by the ACP Trainer (who was selected and payed by the research team), is this the right person to lead this meeting?*<br>*2. Who should be involved during this meeting? The management, the head nurses, the CAP (coordinating advisory physician)? Are these the right people? Which other staff members should be involved?*<br><br>Practical organization<br>*What do you think about the practical organization of this meeting(s)? Is this possible?*<br><br>Infrastructure<br>*Is there room in your NH to carry out such meetings? If not, how could we solve this?*<br><br>Timing/duration<br>*This meeting is organized early, in the first month. Do you feel this is the right time?*<br>*How long do you feel such meeting will take?*<br><br>"Tailoring"<br>*Should there be any changes to this activity to make it more feasible? What would be better how can we make sure it fits your NH? IS it necessary to make it fit?*
**Selection and training ACP Reference persons**<br>Explain	Materials<br>*1. For the selection of the ACP Reference persons the participants in the meeting of the management get a list of criteria and competencies that should support them to select the right staff members. Is this enough? Or do they need more guidance? Is there something specifically missing from this list?*<br>*2. These ACP Reference Persons get a two-day training. During this training*

	they receive the ACP manual for ACP Reference Persons. This includes all documents that are necessary to carry out the intervention (go through documents). What do you feel? Enough? Too much? Too little?<br><br>Procedures<br>*1. What are your thoughts regarding this activity? Regarding the procedures?*<br>*2. What do you think about the procedures to select the ACP Reference Persons?*<br>*3. What do you feel about the training for the ACP Ref Persons? What should be included in the training?*<br><br>Who<br>*1. The trainings are organized by the ACP trainer. Is this the designated person to do so?*<br>*2. Which profile/background do they need? The ACP Reference persons?*<br>*3. Is 1 FTE (full-time equivalent) per 30 beds too much? Enough?*<br><br>Practical organisation<br>*Is a two-day training feasible? Is it possible to free time from the ACP reference persons work schedule to attend these trainings?*<br><br>Infrastructure<br>*These trainings are ideally organized by the nursing home itself. Is there room/private space to do this?*<br><br>Timing/duration<br>*Are these trainings planned at the right time (during the second month of implementation) or is this too late/too early?*<br>*What do you think about these trainings? Are two days too short? Too long?*<br><br>"Tailoring"<br>*Are there any specifics about this training that should be tailored to your nursing home specifically?*
**Part 2**	
**Information session for nursing home staff and general practitioners/GPs (30 min)**<br>Explain	Materials<br>*What do you think of the materials for care professionals and family doctors that will be distributed in the information sessions?*<br><br>Procedures<br>*1. What are your thoughts regarding the procedures of the intervention-activities?*<br>*2. Is an information session the right way to transfer information about ACP (policy and procedures) in the nursing home to these people?*<br><br>Who<br>*1. This information session will be organized by the ACP Trainer, together with*

	the ACP Reference Persons and ideally the CAP. Is there someone else that should be involved?<br>*2. The information session are meant for all NH staff and family physicians. Is this feasible/acceptable? What are your thoughts?*<br><br>Organization<br>*1. These information sessions feasible to organize (In this manner),*<br>*2. IS it possible to free staff from their work schedules, during half an hour? How to organise this?*<br>*4. After all that you've heard, how should we organised his practically?*<br>*5. NH staff is informed about the ACP Reference Persons. How is up to him/her. Is this a good/the best way to do this?*<br>*6. GPs are invited by letter. Is this a good/the best way to do this? IS this feasible?*<br><br>Infrastructure<br>*These sessions will be organised in the participating NHSs? Will there be room to organise such sessions?*<br><br>Timing<br>*1. During month 2, the information sessions ware organized. One for staff and GPs, one for family and residents. If there is no to little response or turn up, the ACP Reference Person can decide to organize more sessions. Do you think his would be feasible within the time span of one month?*<br>*2. The information session should take about 30 minutes. Is this feasible? Enough? Too little/too much?*<br><br>"Tailoring"<br>*1. Is it possible to maybe integrate informational sessions in existing types of informational structures such as family or resident boards, etc.? What do you think? What would be the best way to integrate this?*<br>*2. How does this information session fit daily practice in your NH?*<br>*3. Are there information session that has been organised already in the NH?*
**Information session for residents and family**<br>Explain	Materials<br>*1. What are your thoughts about the materials for residents and family, distributed in the information sessions?*<br><br>Procedures<br>*1. What are your thoughts regarding the procedures of the intervention-activities?*<br>*2. IS the information session the right way to inform residents and family about his?*<br><br>Who<br>*1. This information session can be organized by ACP Trainer, ACP Reference Persons and maybe others? Who?*<br>*2. This information session is organised for all residents and at least one*

	important family member or friend/main contact person in the file of the resident, if the resident is not able to tell him/herself. IS this feasible?<br><br>Organisation<br>*1. Is the organisation of such information sessions feasible?*<br>*2. Is it feasible to get together all residents and at least one of their loved ones together for half an hour?*<br>*3.? After you've heard the envisaged intervention step, is this practically feasible? How would your organised this?*<br>*4. The residents are informed orally or by letter via the ACP Reference Person. Is this feasible? How can we arrange this in a better way?*<br><br>Infrastructure<br>*The information session is held in the NH. Is there any space to organise this?*<br><br>Timing<br>*1.IIs it feasible to organise this in the first or second month of implementation? If there is no turn up, the ACP reference persons can organise additional sessions.*<br>*2. The information session will take about 30 minutes. Is this enough to share this information? Enough? Too many? Too little?*<br><br>"Tailoring"<br>*1. The information sessions are maybe better organised if this is integrated in existing types of information sessions, family or resident boards, etc. Which ones are there? Would this be feasible? Would this be better comparing to what we envisioned?*<br>*2. How does this fit in daily routine care?*<br>*3. Are there any elements that you already do or would like to keep? Are there elements that should definitely be adapted to your local setting?*
**ACP conversations (scheduled)**<br>Explain	Materials<br>*Go through ACP Conversation Guide, Tool and Documents. Ask the participants about their remarks if they did not already say so during the first part of the interview.*<br><br>Procedures<br>*What are your thoughts regarding the procedures of the intervention-activities?*<br><br>Who<br>*1. These ACP Conversations are part 2 and will be organised by mainly the ACP Reference Persons. After training other staff to conversation facilitator they are also doing conversations. IS this feasible?*<br>*2. Ideally these conversations are held with everyone. To make an estimation if someone is still able to be involved in a conversation is a large part of ACP in NHs; but staff should try to involve everyone. Is this feasible/acceptable?*<br>*3. ideally a family member or close loved one is involved in ACP process. The legal representative but in case he or she is not available, someone close to the resident, chosen by the resident. In case the resident has no longer cognitive*

	capacity to tell himself, the family member most involved in the care of the resident is involved in the conversation. IS this feasible/acceptable?<br><br>Organisation<br>*1. The ACP reference Persons make a list of residents of who needs a conversation and who is best to perform the conversation. There is a clear overview of who needs conversations; What do you think of this? Is it better to organised this differently?*<br>*2. After, the conversation facilitator invites the resident and his loved one to the conversation. The conversation facilitator decides how to organise this conversation. Together with the resident/family he decides whether a follow-up conversation (recommended) should be scheduled. Is this feasible/acceptable?*<br>*3. After every conversation held the ACP document has been filled in and the most important decision are documented in the summary. It is recommended to make a copy, send this to the GP and make notes of the destined place in the digital file. IS this practically feasible? How can be optimise this practically? What about digital versus paper ACP documents?*<br><br>Infrastructure<br>*Is there a quite space/enough privacy to schedule such conversations?*<br><br>Timing<br>*1. Is the timing of this organisation these scheduled ACP conversations feasible?*<br>*2. If the resident is only recently admitted is recommended to wait 6 weeks (max 12 weeks) before starting the conversation. Is this feasible/acceptable?*<br>*3. At least every half year there should be a newly scheduled talk if no other triggers were spotted. IS this feasible/acceptable?*<br><br>“Tailoring”<br>*1. How are you practically organising ACP in tis NH?*<br>*2. Are there elements about this envisaged intervention that should be held free to organise by the NHs themselves?*
**Training to recognize signals (1u30min)**<br>Explain	Materials<br>*Not applicable. If you haven’t discussed the informational leaflet for staff and GPs yet, do it here.*<br><br>Procedures<br>*1. What are your thoughts regarding the procedures of the intervention-activities?*<br>*2. What are your thoughts about these trainings sessions? Is this useful?*<br><br>Who<br>*1. Is it possible to invite care assistants, cleaning staff or others? Or only care assistants?*<br>*3. Would it be possible to the free people from their work schedules to follow this training? How much time is available?*

	4. Are there other (care) professionals that should also be involved in these trainings, making sure they can schedule ACP conversations?<br>*5. This training is organised and preferably given by the ACP trainers assisting the ACP Reference Persons. IS this feasible/acceptable?*<br>*6. Ideal groups will be 10 top 15 people? Should we divide them in groups by function? Or mix them?*<br>*7. Are ACP Reference Persons up for motivating the other staff? Would we make this obligatory part of their mission/task?*<br><br>Organisation<br>*1. The ACP Reference Persons are in charge to organise these trainings practically, with the support of the ACP Trainer? How can this best be organised according to you?*<br>*2. The ACP reference persons make sure all staff is informed about these sessions and all have the chance to participate. IS this feasible/acceptable?*<br><br>Infrastructure<br>*Is the right infrastructure available in every NH?*<br><br>Timing<br>*1. The training will take up at least 11/2 hours. Too short/too long?*<br>*2.IIs it possible to free staff from their work schedules?*<br><br>“Tailoring”<br>*1. How to integrate these trainings into daily routine care? What do you recommend?*<br>*2. Are there trainings that are already organised? Are there elements that definitely should be adjusted or for the NH to be free to adjust to fit their practice better?*
**Training to carry out ACP conversations (3 hours**)<br>Explain	Materials<br>*Discuss information materials and ACP conversation documents.*<br><br>Procedures<br>*What are your thoughts regarding the procedures of the intervention-activities? Are these trainings useful?*<br><br>Who<br>*1. Appropriate for nursing staff? All? How many are there?*<br>*2. Appropriate for volunteers? Which ones? How many are there?*<br>*3. Can nurses be freed from their time to follow these trainings? How much time maximum?*<br>*4. Are there other staff members that should follow these trainings to conduct ACP conversations?*<br>*5. Training is organised by the ACP Trainer and ACP Reference persons? What are your thoughts?*<br>*6. Ideally in groups of 10 or 15? Mix or split up according to profession?*<br>*7. Is staff motivated to get involved in these trainings or should we make them*

	obligatory?<br><br>Organisation<br>*1. ACP Reference persons are in charge to organize these trainings? They get support from the ACP Trainer in the beginning? Is this feasible?*<br>*2. The ACP reference persons makes sure all staff and volunteers are aware of all training sessions. How to do this?*<br><br>Infrastructure<br>*Is there appropriate space available in all nursing homes?*<br><br>Timing:<br>*1. Training takes about 3 hours. Too long/short?*<br>*2. Is it possible to free nursing staff for a time span of 3 hours from their work schedules? All at the same time?*<br><br>"Tailoring":<br>*Does this fit in your daily practices? What should be adapted? Are there already elements that exist now?*
**Reflection sessions (e.g. reflective debriefing)**<br>Explain	Materials<br>*What about the reflection instrument? Is this useful?*<br><br>Procedures:<br>*What are your thoughts regarding the procedures of the intervention (e.g. One every 3 months the reflection sessions are organised about the past 3 months)? Useful?*<br><br>Who<br>*1. These reflection sessions are about the past 3 months and ideally about residents that have passed away. Is this feasible? How many residents die each 3 months?*<br>*2. Who should be involved in these sessions? We think about all care staff involved in the care of the residents that have died in the past 3 months.*<br>*3. Sessions are organized by the ACP Reference persons, together with the ACP Trainer in the beginning.*<br><br>Organisation<br>*The ACP References persons are free to decide how to organise these sessions. What would be feasible/acceptable?*<br><br>Infrastructure:<br>*Is there a private room/space available?*<br><br>Timing:<br>*Once every 3 months? Is this too much? It can take around one hour but can vary according to the number of deaths in the past 3 months.*

	"Tailoring"<br>*How does this fit in the regular daily care practice? Are you already organizing types of reflection? Can this be integrated in existing meetings types? How? Which elements could differ from NH to NH?*
**Monthly Multidisciplinary Meetings (MDO's)**<br>Explain	Materials<br>*Discuss ACP document which can be used to inform staff about (changes in) wishes and preference of resident/family and can be taken to MDO?*<br><br>Procedures<br>*1. Monthly MDO discussing all residents and all past and future ACP?*<br>*2. How to integrate ACP conversations and outcomes of these conversations in the MDO. Would this be useful to inform other staff about ('changes in) wishes and preferences of resident and family? Is this feasible to integrate in MDO? Or better to be integrated in other type of meeting? Or not at all?*<br><br>Who<br>*Who should be involved in such an MDO? Should the GP be involved too? How are you planning on making sure the GP is informed about (changes) in wishes and preferences of the resident/family?*<br><br>Organisation<br>*It is free to decide how to organise these meetings? How would you recommend to organise an MDO practically?*<br><br>Infrastructure<br>*same as above*<br><br>Timing<br>*An MDO is normally organised every month? How much are you organising this type of meeting? How long does such meeting last?*<br><br>"Tailoring"<br>*same as above*
**Part 3**	
	In the final stages the ACP trainers support decreases. What is your preferred mode of contact (by phone? Drop by?) When should he/she best drop by the nursing home?
**Audit**<br>Explain	Materials<br>*Discuss audit instrument.*<br><br>Procedures<br>*1. What are your thoughts regarding the procedures of the intervention-activities?*<br>*2. Do you feel such audit meeting is useful?*

	Who<br>*1. This audit is organized by ACP Reference Persons, quality coordinator (if available), management, head nursing staff and CAP. Anyone else?*<br>*2. Should results be reported to other staff? How and who?*<br><br>Organisation<br>*Organised at least once a year/yearly. How?*<br><br>Infrastructure<br>*NA*<br><br>Timing<br>*How much time will it take to do this exercise and meet with everyone to discuss and make plans for improvement?*<br><br>“Tailoring”:<br>*same as above*
